# Novel metabolite madeirone and neomarinone extracted from *Streptomyces aculeoletus* as marine antibiofilm and antifouling agents

**DOI:** 10.3389/fchem.2024.1425953

**Published:** 2024-07-25

**Authors:** Julian L. Wissner, Joana R. Almeida, Inês R. Grilo, Jhenifer F. Oliveira, Carolina Brízida, Wendy Escobedo-Hinojosa, Panayiota Pissaridou, Marlen I. Vasquez, Isabel Cunha, Rita G. Sobral, Vítor Vasconcelos, Susana P. Gaudêncio

**Affiliations:** ^1^ Associate Laboratory i4HB, Institute for Health and Bioeconomy, NOVA Faculty of Sciences and Technology, NOVA University of Lisbon, Lisbon, Portugal; ^2^ UCIBIO, Applied Molecular Biosciences Unit, Chemistry and Life Sciences Departments, NOVA Faculty of Sciences and Technology, NOVA University of Lisbon, Lisbon, Portugal; ^3^ Unidad de Química en Sisal, Facultad de Química, Universidad Nacional Autónoma de México, Yucatán, Mexico; ^4^ CIIMAR/CIMAR—Interdisciplinary Centre of Marine and Environmental Research, University of Porto, Terminal de Cruzeiros do Porto de Leixões, Matosinhos, Portugal; ^5^ Department of Chemical Engineering, Cyprus University of Technology, Limassol, Cyprus; ^6^ Biology Department, Faculty of Sciences, Porto University, Porto, Portugal

**Keywords:** blue biotechnology, marine natural products, actinomycetes bioprospection, meroterpenoids, hybrid isoprenoids, marine biofilm and biofouling, antifouling. eco-friendly paints and coatings

## Abstract

**Introduction:** Biofouling poses a significant economic threat to various marine industries, leading to financial losses that can reach billions of euros annually. This study highlights the urgent need for effective alternatives to traditional antifouling agents, particularly following the global ban on organotin compounds.

**Material and methods:** *Streptomyces aculeolatus* PTM-346 was isolated from sediment samples on the shores of the Madeira Archipelago, Portugal. The crude extract was fractionated using silica flash chromatography and preparative HPLC, resulting in two isolated marinone compounds: madeirone (**1**), a novel marinone derivative discovered in this study, and neomarinone (**2**). The antifouling activities of these compounds were tested against five marine bacterial species and the larvae of the mussel *Mytilus galloprovincialis*. Additionally, *in silico* and *in vivo* environmental toxicity evaluations of madeirone (**1**) and neomarinone (**2**) were conducted.

**Results:** Madeirone (**1**) demonstrated significant antibiofilm efficacy, inhibiting *Phaeobacter inhibens* by up to 66%, *Marinobacter hydrocarbonoclasticus* by up to 60%, and *Cobetia marina* by up to 40%. Neomarinone (2) also exhibited substantial antibiofilm activity, with inhibition rates of up to 41% against *P. inhibens*, 40% against *Pseudo-oceanicola batsensis*, 56% against *M. hydrocarbonoclasticus*, 46% against C. marina, and 40% against *Micrococcus luteus*. The growth inhibition activity at the same concentrations of these compounds remained below 20% for the respective bacteria, highlighting their effectiveness as potent antibiofilm agents without significantly affecting bacterial viability. Additionally, both compounds showed potent effects against the settlement of *Mytilus galloprovincialis* larvae, with EC_50_ values of 1.76 µg/mL and 0.12 µg/mL for compounds (**1**) and (**2**), respectively, without impairing the viability of the targeted macrofouling species. *In silico* toxicity predictions and *in vivo* toxicity assays both support their potential for further development as antifouling agents.

**Conclusion:** The newly discovered metabolite madeirone (**1**) and neomarinone (**2**) effectively inhibit both micro- and macrofouling. This distinct capability sets them apart from existing commercial antifouling agents and positions them as promising candidates for biofouling prevention. Consequently, these compounds represent a viable and environmentally friendly alternative for incorporation into paints, primers, varnishes, and sealants, offering significant advantages over traditional copper-based compounds.

## 1 Background

Biofouling is a common biological phenomenon involving the adhesion of micro and macroorganisms, such as barnacles and mussels, to aquatic submerged surfaces (Magin et al., 2010). This occurrence poses a serious threat to maritime industries, resulting in significant economic losses and adverse impacts on marine environments and the economy (Xu et al., 2010). It causes substantial financial burdens, amounting to billions of euros annually, affecting aquaculture, shipping, and other industries dependent on coastal and offshore infrastructures. As ocean warming increases, the task of managing marine biofilms and biofouling is becoming more challenging (Schultz et al., 2011; Conrad and Poling-Skutvik, 2018; Sushmitha et al., 2023). Tributyltin was utilized to prevent biofouling, yet it resulted in serious environmental issues due to its toxicity, being banned by the International Maritime Organization in 1990 (Sonak et al., 2009). The global prohibition of organotin compounds as antifouling agents has amplified the urgency for safe and effective alternatives. Therefore, the identification of environmentally friendly strategies is imperative. Currently, there is a lack of sustainable, cost-effective, and environmentally benign solutions to adequately address this challenge. The quest for an environmentally safe antifouling agent is particularly noteworthy due to the persistent impact of biofoulers on marine habitats and the detrimental effects of biocides on the environment. Recent research efforts have concentrated on isolating natural, eco-friendly antifouling agents to counteract the toxicities associated with synthetic counterparts (Callow and Callow, 2011).

The marine ecosystem has proven to be a fundamental reservoir of economically and biotechnologically significant secondary metabolites. When exploring novel bioresources for economically important products, the marine environment garners special attention owing to its extraordinary diversity and extreme conditions, acknowledged for generating metabolites of immense value. It stands as an untapped resource for uncovering innovative secondary metabolites with diverse potential, as well as a wide array of bioactive compounds suitable for various biotechnological applications (Kirschner and Brennan, 2012; Rotter et al., 2021).

Marine-derived actinomycetes have emerged as a valuable source for such secondary metabolites that have demonstrated their importance for industries, supported by research on their properties and versatile applications (Bérdy, 2005). Notably, these actinomycetes produce a diverse collection of active metabolites, some of which exhibit remarkable antifouling properties. To date, there are very few studies on actinomycetes reporting antibiofilm activity against both Gram-positive and -negative bacteria, especially coupled with effects inhibiting larval settlement and the acetylcholinesterase enzyme, which signifies robust anti-macrofouling activity (Gaudêncio and Pereira, 2022; Morgan et al., 2023).

A comprehensive bibliographic search was conducted on antibiofilm and antifouling natural products from actinomycetes (Xu et al., 2007; Xu et al., 2010; Selvin, 2009; Cho, 2012; Cho and Kim, 2012; Cho et al., 2012; Prakash et al., 2015; Gopikrishnan et al., 2016; Waturangi et al., 2017; Kavitha and Vimala, 2020; Pereira et al., 2020; She et al., 2022; Stalin et al., 2022). Surprisingly, the reported studies referred to *Streptomyces*, with only one exception. Among actinomycetes, the *Streptomyces* genus has proven particularly prolific in producing antifouling agents. The chemical groups of these compounds encompass terpenoids, polyketides, furanones, butenolides, glycoglycerolipids, and alkaloids (Xu et al., 2010; Cho, 2012; Morgan et al., 2023).

For instance, *Streptomyces praecox* 291-11, isolated from *Undaria pinnatifida* rhizosphere, produced diketopiperazines with antifouling activity against *Ulva pertusa* and *Navicula annexa* (Cho et al., 2012). *Streptomyces coelescens* PK206-15, associated with seaweed, produced glycoglycerolipids inhibiting various fouling organisms (Cho, 2012). *Streptomyces cinnabarinus* PK209, co-cultured with *Alteromonas* sp. KNS-16, produced lobocompactol with significant antifouling activity (Cho and Kim, 2012). *Streptomyces chrestomyceticus* BCC 24770I synthesized albofungins, exhibiting antibiofilm and anti-macrofouling activities (She et al., 2022). Moreover, *Streptomyces thermolineatus* VITKV6A produced oxycyclopentadien with antimicrofouling activity against biofilm-forming bacteria (Stalin et al., 2022). *Streptomyces dendra* sp. nov. MSI051, isolated from *Dendrilla nigra*, demonstrated antagonistic potential against biofilm bacteria (Selvin, 2009). A deep-sea *Streptomyces* strain inhibited *Balanus amphitrite* larval settlement (Xu et al., 2007). Additionally, *Streptomyces fradiae* PE7 from Vellar estuarine sediment reported antifouling activity with quercetin (Gopikrishnan et al., 2016). *Streptomyces fradiae* RMS-MSU, isolated from Manakkudy mangroves, displayed antagonistic activity against marine biofilm bacterial strains (Prakash et al., 2015).

Additionally, antibiofilm and antifouling activity of napyradiomycins isolated from *Streptomyces aculeolatus* (*S. aculeolatus*), was reported by Gaudêncio and co-workers (Pereira et al., 2020).

The discovery of molecules exhibiting antibiofilm properties without antimicrobial effects presents an important alternative for treating infections linked to microorganisms that form biofilms. These compounds impede biofilm formation, thereby exposing bacteria to the surrounding environment, all without applying the typical selective pressure that often triggers the emergence of resistance mechanisms (Ahmad et al., 2014; Bauermeister et al., 2019; Sabotič et al., 2024).

Based on the aforementioned considerations, our research focused on investigating the marine-derived *S*. *aculeolatus* PTM-346 to evaluate the produced secondary metabolites as inhibitors of both marine micro and macrofouling.

## 2 Methods

### 2.1 Marine-derived actinomycetes isolation from ocean sediments

In June 2012, sediment samples were gathered off the shores of the Madeira Archipelago, Portugal (Prieto-Davó et al., 2016). Strain PTM-346 was isolated from samples retrieved at a depth of 14 m through SCUBA diving in Madeira Island waters. The sediment processing involved a heat-shock method: approximately 0.5 g of wet sediments were mixed with 2 mL of sterile seawater (SSW), settled briefly, and then subjected to a 6 min heat treatment at 55°C. Subsequently, 50 µL of the upper layer was spread on an agar plate containing seawater-based medium SW (1.8% p/v agar), supplemented with the antifungal cycloheximide (100 μg/L). The plates were incubated at room temperature (approximately 25°C) and regularly monitored for actinomycete growth over a period of 6 months. PTM-346 was sequentially transferred to fresh seawater-based A1 medium (10 g starch, 4 g yeast extract, 2 g peptone per L) until obtaining a pure strain. Strain PTM-346 was cultivated in A1 liquid culture medium (without agar) and preserved by cryopreservation in 10% (v/v) glycerol at −80°C.

### 2.2 PTM-346 actinomycete strain phylogenetic characterization

The actinomycete PTM-346 used in this study is phylogenetically related to the species *S*. *aculeolatus*, previously isolated by our group from oceanic sediments from the Madeira Archipelago (Prieto-Davó et al., 2016). The phylogenetic analysis involved incubating the culture in 20 mL of A1 medium with agitation (200 rpm) at 25°C for a duration of 7 days. Genomic DNA extraction was carried out using the Wizard^®^ Genomic DNA Purification Kit (Promega, Madison, WI, United States) according to the protocol adapted for Gram-positive bacteria. To ensure sufficient genomic DNA extraction, extended incubation periods with lysozyme and RNase solution were employed, aligning with the manufacturer’s recommendations. The 16S rRNA gene was then amplified using the universal primers 27F (5′-AGA​GTT​TGA​TCC​TGG​CTC​AG-3′) and 1492R (5′-TAC​GGC​TAC​CTT​GTT​ACG​ACT​T-3′) (Gontang et al., 2007; Prieto-Davó et al., 2016; Pinto-Almeida et al., 2022). Subsequently, the amplified products were purified using the SureClean PCR cleanup kit (BioLine, London, UK) following the provided protocol. The purified PCR products underwent cycle sequencing at STABVIDA, Lda (www.stabvida.net), utilizing the ABI BigDye^®^ Terminator v3.1 Cycle Sequencing Kit (Needham, MA, United States). The resulting sequences were analyzed on an ABI PRISM^®^ 3730xl Genetic Analyzer (Needham, MA, United States), and the sequence traces were edited using Sequencing Analysis 5.3.1 from Applied Biosystems™ (Needham, MA, United States). For sequence comparison with the GenBank database, the BLASTn algorithm was employed (Altschul et al., 1990). The PTM-346 sequence has been deposited in GenBank under the accession number KP869060.1 and is accessible at www.ncbi.nlm.nih.gov/genbank.

### 2.3 Culture conditions for actinomycete PTM-346 crude extract production

The actinomycete strain PTM-346 was cultured in 40 Erlenmeyer flasks of 2 L volume, each with 1 L of seawater-based A1 medium. The cultures were agitated at 200 rpm and incubated at a temperature of 30°C. After 15 days of incubation, the culture underwent three extractions using half the volume of ethyl acetate (EtOAc) each time. The resulting mixture was then evaporated under vacuum, resulting in the production of approximately 6.5 g of crude extract.

### 2.4 Actinomycete PTM-346 secondary metabolites isolation and structure elucidation

PTM-346 crude extract, approximately 6.5 g in quantity, underwent fractionation using silica flash chromatography. This process involved step gradients of isooctane/EtOAc, followed by EtOAc/MeOH. The secondary metabolites (**1**) and (**2**) from PTM-346 were successfully obtained in the 4:6 and 0:1 fraction of isooctane/EtOAc, respectively. Further isolation procedures were carried out through reversed-phase HPLC, utilizing a Phenomenex Luna column (250 mm × 4.6 mm, 5 μm, 100 Å) at a flow rate of 1.5 mL/min, with DAD (Diode Array Detector) 190–500 nm. A gradient solvent system, ranging from 10% to 100% H_2_O:ACN (0.1% trifluoroacetic acid) over a 40 min period, followed by 100% acetonitrile during additional 40 min enabled the isolation of compound (**1**) (3.2 mg, yellow powder). Wilts, a gradient solvent system, ranging from 10% to 100% H_2_O:ACN in (0.1% trifluoroacetic acid) over a 20 min period, followed by 100% acetonitrile during additional 31 min allowed the isolation of compound (**2**) (3.9 mg, yellow powder). Infrared (IR) spectra were obtained using a Perkin Elmer Spectrum Two FT-IR Spectrometer. High-resolution ESI-TOF mass spectra were acquired through services provided by the mass spectrometry facility at the Department of Chemistry and Biochemistry, University of California, San Diego, La Jolla, CA, using an Agilent 6,230 Accurate-Mass TOFMS spectrometer in negative mode. Low-resolution LC/MS data were measured at NOVA-FCT analysis Lab, Portugal, utilizing an Agilent 1,200 Series LC with Binary pump HPLC System coupled with a Mass Spectrometry Agilent 6130B Single Quadropole (API-ES source, 3000 V), in positive mode. The analysis employed a reversed-phase C_18_ column (Phenomenex Luna, 100 mm × 1.0 mm, 5 µm), utilizing an H_2_O:ACN 10%–100% gradient with 0.1% formic acid at a flow rate of 0.7 mL/min, during 30 min. Elemental analysis (EA) was performed using a analyzer Thermo Finnigan-CE Instruments Flash EA 1112 CHNS series, in duplicate at NOVA FCT, Chemistry Department Analytical Lab. ^1^H-, ^13^C- and 2D- NMR spectral data measurements were conducted at 400 or 100 MHz using Bruker Advance and Bruker BioSpin spectrometers, (Ettlingen, Germany. Tetramethylsilane (TMS) was used as an internal standard. CDCl_3_ and DMSO-d6 were used as solvents for compounds (**1**) and (**2**), respectively.

Compound (**1**): yellow solid (3.2 mg); **Rt** = 50.1 min; **UV λ**_**max**_ **(nm):** 210, 250, 290, and 335; **IR NaCl ν**_**max**_ **(cm** ^**−1**^ **):** 3273.34, 2962.53, 2926.36, 1618.95, 1574.85, 1440.33, 1242.54, 1048.52, 1085.93; **EA:** C, 73.61: H, 7.60: O, 18.86, empirical formula C_25_H_28_O_6_; **MS** *m/z* **:** 425.2 Da [M + H]^+^, 849.3 Da [2M + H]^+^, (424.22 calcd. for C_26_H_32_O_5_); ^**13**^ **C-NMR (100 MHz, CDCl**_**3**_ **)** δ_C_, 183.8, 180.1, 161.0, 159.3, 156.8, 139.1, 132.8, 128.9, 124.6, 111.2, 109.5, 109.3, 88.0, 56.3, 46.7, 40.3, 33.3, 31.7, 31.1, 27.1, 25.6, 21.2, 20.0, 19.1, 15.9, 15.3 ppm; ^**1**^ **H-NMR (400 MHz, CDCl**_**3**_ **)** δ_H_ 7.14 (s, 1H), 5.96 (s, 1H), 5.33 (s, 1H), 4.78 (q, 6.6 Hz, 1H), 3.76 (s, 2H), 1.95–1.78 (m, 4H), 1.68 (m, 1H), 1.46 (s, 3H) 1.37 (d, 6.6 Hz, 3H), 1.32 (m, 2H), 1.24–1.14 (m, 5H), 0.74 (m, 3H), 0.72 (s, 3H) ppm. The obtained spectrometric and spectroscopic data suggest a novel marinone derivative.

Compound (**2**): yellow solid (3.9 mg); **Rt** = 34 min, **UV λ**_**max**_ **(nm):** 218, 263, 312, 400 nm; **IR NaCl ν**_**max**_ **(cm** ^**−1**^ **):** 2957.52, 2923.03, 2851.87, 1738.95, 1572.27, 1464.32, 1379.87, 1303.05, 1182.50, 1052.63 cm^-1^; **HR-MS** *m/z* **:** 424.225 calcd. for C_26_H_32_O_5_, measured 424.2249; **MS:** *m/z* 425.2 Da [M + H]^+^; 849.3 Da [2M + H]^+^; ^**13**^ **C-NMR (100 MHz, DMSO-d6):** δ_C_ 183.2, 181.1, 159.7, 157.2, 152.6, 138.7, 130.6, 127.1, 123.3, 120.0, 107.9, 107.6, 86.1, 46.5, 40.3, 32.2, 30.5, 30.0, 26.1, 24.7, 20.3, 19.0, 18.2, 15.1, 14.4, 7.6 ppm; ^**1**^ **H-NMR (400 MHz, DMSO-d6):** δ_H_ 7.03 (s, 1H), 5.34 (s, 1H), 4.79 (q, 6.6 Hz, 1H), 1.98 (s, 3H), 1.93–1.82 (m, 4H), 1.67 (m, 1H), 1.47 (m, 3H), 1.37 (d, 6.6 Hz, 3H), 1.34 (m, 2H), 1.19 (m, 5H), 0.75 (m, 3H), 0.73 (s, 3H) ppm. The obtained spectrometric and spectroscopic data is in accordance with the reported for metabolite neomarinone (Hardt et al., 2000; Kalaitzis et al., 2003).

### 2.5 Antibiofilm activity (microfouling) evaluation

#### 2.5.1 Marine fouling bacteria culture conditions

To assess antimicrofouling activity, we selected five marine bacterial species as models: *Marinobacter hydrocarbonoclasticus* DSM 8798 (ATCC 49840), *C. marina* DSM 4741, *Phaeobacter inhibens* DSM 17,395, *Pseusooceanicola batsensis* DSM 15,984, and *Micrococcus luteus* DSM 20030 (ATCC 4698) (Michael et al., 2016). These strains, sourced from DSMZ (Leibniz Institute DSMZ—German Collection of Microorganisms and Cell Cultures), were cultured in liquid marine broth (Carl Roth GmbH, Karlsruhe, Germany) with agitation at 180 rpm or on agar-supplemented marine broth. Incubation temperatures were set at 28°C for *M. hydrocarbonoclasticus* and *Cobetia marina*, 30°C for *P. inhibens* and *P. batsensis*, while *M. luteus* was maintained at 37°C in Brain Heart Infusion broth (BHI, Becton Dickinson, GmbH, Heidelberg, Germany) under similar agitation conditions.

#### 2.5.2 Antibacterial activity evaluation

The antibacterial efficacy of compounds (**1**) and (**2**) was assessed in 96-well polystyrene flat-bottom microplates (Nunclon Delta Surface, Thermo Scientific, Roskilde, Denmark) following established procedures (Bauermeister et al., 2019). In the initial screening, bacterial overnight cultures were diluted to an optical density (OD_600_nm) of 0.2 and incubated statically at appropriate temperatures. The cultures were treated with the following final concentrations per well: 31.25, 15.60, 7.81, 3.91, 1.95, and 0.98 μg/mL (2-fold serial dilutions) of the compounds, solubilized in DMSO, or left untreated. After 24 h (*M. hydrocarbonoclasticus*, *C. marina*) or 48 h (*P. batsensis*, *P. inhibens*, *M. luteus*) incubation, optical density at 600 nm (OD_600_) was measured using a Molecular Devices Spectra Max 190. Growth inhibition percentages were calculated relatively to untreated bacterial species, using DMSO as a negative control. CuSO_4_ (0.16 μg/mL), a recognized antifouling agent, was used as positive control. All assays were conducted in triplicate, and the results represent the mean and standard error of the mean (SEM). Statistical analysis was carried out using GraphPad Prism 8.0.2 (San Diego, CA, United States), employing one-way ANOVA followed by Dunnett’s multiple comparisons test against the negative control.

#### 2.5.3 Antibiofilm activity evaluation

The antibiofilm potential of compounds (**1**) and (**2**) against the five marine bacterial species was investigated in 96-well polystyrene flat-bottom microplates (Nunclon Delta Surface, Thermo Scientific, Roskilde, Denmark), as described previously (Bauermeister et al., 2019; Pereira et al., 2020). In the initial screening, bacterial overnight cultures were diluted to an OD_600_ of 0.2 and incubated statically at appropriate temperatures. The cultures were tested at 31.25, 15.60, 7.81, 3.91, 1.95, 0.98 μg/mL concentrations (2-fold serial dilutions) of compounds (**1**) and (**2**), solubilized in DMSO, or left untreated. After 24 h (*M. hydrocarbonoclasticus*, *C. marina*) or 48 h (*P. batsensis*, *P. inhibens*, *M. luteus*) incubation, OD_600_ was measured. The planktonic cells and media were discarded, and the wells were washed twice with deionized water. Biofilms were fixed, stained, and quantified by measuring the OD_600_ after solubilization with acetic acid. Biofilm inhibition percentages were calculated relative to untreated bacterial species, using DMSO as a negative control and CuSO_4_ (0.16 μg/mL), as a positive control. All assays were performed in triplicate, and the results represent the mean and standard error of the mean (SEM). Statistical analysis was conducted using GraphPad Prism 8.0.2, employing one-way ANOVA followed by Dunnett’s multiple comparisons test against the negative control.

### 2.6 Antibiofouling activity (macrofouling) evaluation

#### 2.6.1 Mussel larvae (*Mytilus galloprovincialis*) acute toxicity assay

The *in vivo* antimacrofouling activity of compounds (**1**) and (**2**) was evaluated against mussel *M. galloprovincialis* adhesive larvae (plantigrades) in an acute bioassay. Juvenile mussel aggregates were harvested from the intertidal rocky shore during low spring tides at Memória beach, Matosinhos, Portugal. In the laboratory, precisely before the bioassays, mussel plantigrade larvae were meticulously screened and isolated from the juvenile aggregates using a binocular microscope (Olympus SZX2-ILLT, Hamburg, Germany). The isolated larvae were then washed with filtered seawater to eliminate organic debris, and competent plantigrade larvae, characterized by foot exploratory behavior, were selected for the exposure bioassays, following established protocols (Almeida et al., 2017; Almeida et al., 2020; Antunes et al., 2019; Mabrouk et al., 2020). Plantigrades were exposed in 24-well polystyrene plates for 15 h in darkness at 18°C. DMSO served as the solvent for the testing compounds in stock and working solutions. The DMSO concentration in the tested solutions was consistently maintained at 0.1%. Each condition was replicated in four wells, with five larvae per well. Two negative controls, one with ultra-pure water and other with DMSO were included in all bioassays, along with a positive control using 0.16 μg/mL CuSO_4_, as a reference antifouling agent. The anti-settlement bioactivity was assessed based on the presence or absence of fixed byssal threads produced by each individual larva for all tested conditions. Compounds (**1**) was tested at successive concentrations 10, 5, 2.5, 1.2, and 0.6 μg/mL and compound (**2**) at 4, 2, 1, 0.5, 0.25, 0.12 μg/mL to determine the semi-maximum response concentrations (EC_50_) with an anti-settlement effect on mussel larvae. EC_50_ values for each compound were calculated using Probit regression analysis. Significance was considered at *p* < 0.01, and 95% lower and upper confidence limits (95% LCL; UCL). The software IBM SPSS Statistics 28 was used for statistical analysis. The therapeutic ratio (LC_50_/EC_50_) was employed to evaluate the effectiveness *versus* the toxicity of the compounds (Qian et al., 2009; Almeida and Vasconcelos, 2015).

### 2.7 *In silico* environmental toxicity evaluation

The evaluation of *in silico* toxicity was conducted using the Toxicity Estimation Software Tool (T.E.S.T.) version 5.1.2 (CCTE, EPA, 2022), available at https://www.epa.gov/chemical-research/toxicity-estimation-software-tool-test.

### 2.8 *In vivo* environmental toxicity evaluation

The *in vivo* evaluation of compounds (**1**) and (**2**) for acute toxicity was performed towards marine bacterium *Aliivibrio fischeri,* and planktonic crustacean *Daphnia magna.* The chronic ecotoxicity tests were evaluated using algae *Pseudokirchneriella subcapitata and Phaeodactylum tricornutum.*

The inhibition of bioluminescence of the marine bacterium *A. fischeri*, were studied following a micro adaptation of the standard procedure ISO 11348-3 (“Water Quality-Determination of the inhibitory effect of water samples on the light emission of *Vibrio fischeri -* Luminescent bacteria test–using freeze-dried bacteria method”). Briefly, frozen lyophilized bacterial cells (Abraxis, Warminster, PA, United States) were reconstituted in Reconstitution Solution, supplied by the manufacturer of the Abratox kit (Abraxis). Madeirone (1) and neomarinone (2) were dissolved in DMSO and tested at the concentrations of 100, 50, 25, 12.5, 6.25, 3.13, and 1.56 μg/mL. The emitted luminescence was measured using a luminometer at time 0 min and at different exposure times (5, 15 and 30 min) and the percentage of inhibition of bioluminescence was determined. Each experiment was performed in duplicate and the results of three independent experiments with <10% SD are presented. Phenol was used as a positive control.

The immobilization of *D. magna* at 24 h and 48 h was studied following a micro adaptation of the OECD Guideline 202 procedures (“*Daphnia* sp., Acute Immobilisation Test and Reproduction Test”), using the Daphtoxkit F (Microbiotests, Gent, Belgium). Firstly, the ISO matrix medium was prepared, aerated and adjusted to pH 7. Next, the neonate daphnids were incubated for 72 h at 20 ± 1°C with light and fed spirulina 2 h prior to use, according to the manufacturer’s instructions. Subsequently, the daphnids were exposed to madeirone (1) and neomarinone (2) dissolved in DMSO at the concentrations of 100, 50, 25, 12.5, 6.25, 3.13, and 1.56 μg/mL, and the immobilization of the daphnids was recorded by direct observation at 24 and 48 h. For the miniaturization of the procedure, one daphnid neonate was exposed to each compound in 10 mL sterile tubes containing 2 mL of ISO matrix medium, with the compounds at the determined concentrations. The assays were performed in triplicate. Potassium dichromate (K_2_Cr_2_O_7_) was used as a positive control.

Freshwater and marine algal growth inhibition was assessed using *P. subcapitata and P. tricornutum* according to OECD Guideline 201 procedures (“Alga, Growth Inhibition Test”), ISO-8692 (“Water quality–Fresh water algal growth inhibition test with unicellular green algae”) and ISO-10253. Briefly, the algae cells were de-immobilized from algal beads using Matrix dissolving medium (Microbiotests), according to the manufacturer’s instructions, and grown in flasks containing algal culturing medium which was prepared following the OECD Guideline 201 and ISO-8692. Flasks with algae were kept for 7 days under light with orbital agitation. When the algae culture had sufficient cells, as verified by the optical density at 670 nm, these were tested at an initial cell density of 1 × 10^6^ cells/mL in algal culturing medium. Madeirone (1) and neomarinone (2) were dissolved in DMSO and tested at the concentrations of 100, 50, 25, 12.5, 6.25, 3.13, and 1.56 μg/mL. The algae were cultured in microplates in triplicate and were kept for 4 days under light with orbital agitation. The optical density was measured at 670 nm, at time zero and after 24, 48, 72 and 96 h incubation, determining the algal growth inhibition of the compounds. Each experiment was performed in triplicate and the results of two independent experiments with <10% SD are presented. Growth inhibition calculations were performed using cell numbers (biomass), as determined by a calibration line. Potassium dichromate (K_2_Cr_2_O_7_) was used as a positive control.

## 3 Results and discussion

### 3.1 Structural elucidation of marinones isolated from *Streptomyces aculeolatus* PTM-346

Marine-derived strain PTM-346 was taxonomically characterized as *S*. *aculeolatus* (Prieto-Davó et al., 2016). These species belong to MAR4 group, which are known to produce meroterpenoids (Murray et al., 2020) from the classes napyradiomycins, marinones, lavanducyanins, nitropyrrolines, novobiocins or chlorobiocins (Pathirana et al., 1992; Hardt et al., 2000; Kawasaki et al., 2006; Gallagher et al., 2010; Gallagher et al., 2013; Kirschner and Brennan, 2012; Gallagher and Jensen, 2015).

In this study, the ethyl acetate (EtOAc) extracts of *S*. *aculeolatus* PTM-346 (Prieto-Davó et al., 2016; Bauermeister et al., 2019) underwent micro and macro antifouling bioassay-directed fractionation and isolation. This process initially was performed using silica flash chromatography and subsequently employed C_18_ reversed-phase semi-prep HPLC, resulting in the isolation of two compounds (**1**, **2**). The chemical structures of (**1**) and (**2**) were elucidated using MS spectrometry and the interpretation of UV-Vis, IR, 1D- and 2D-NMR spectroscopic data. Both isolated compounds were determined to belong to the class of marinones, which are hybrid isoprenoids, sesquiterpene naphthoquinones with a mixed origin of polyketides and terpenoids also designated meroterpenoids (Olano et al., 2008; Gallagher et al., 2013). To date, six compounds of the marinone class have been reported (Table 1).

**TABLE 1:** List of all marinone metabolites described in the literature (Pathirana et al., 1992; Hardt et al., 2000; Kalaitzis et al., 2003).

Compound	Molecular formula	Chemical structure	*m/z* (Da)	Ref.
Marinone	C_25_H_27_BrO_5_	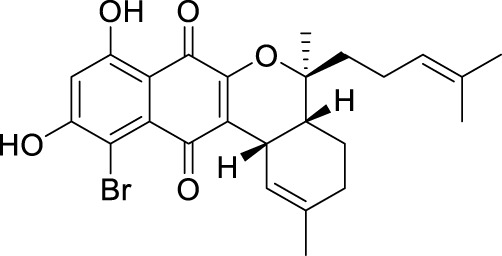	487.4	Pathirana et al. (1992)
Debromomarinone	C_25_H_28_O_5_	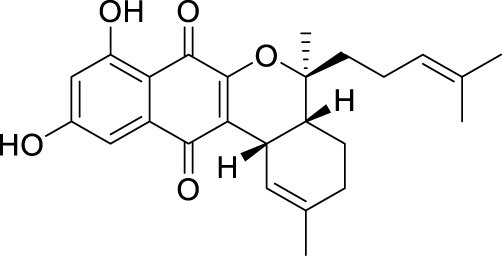	408.5	Pathirana et al. (1992)
Isomarinone	C_25_H_27_BrO_5_	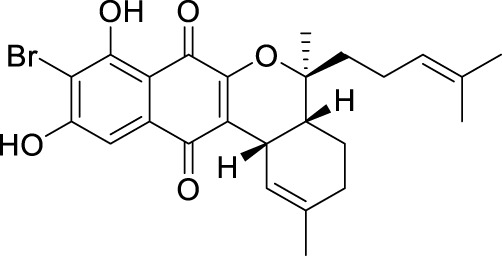	487.4	Hardt et al. (2000)
Hydroxydebromomarinone	C_25_H_28_O_6_	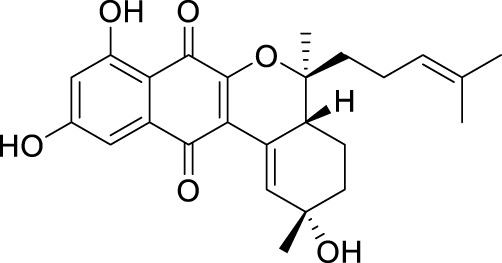	424.5	Hardt et al. (2000)
Metoxydebromomarinone	C_26_H_30_O_6_	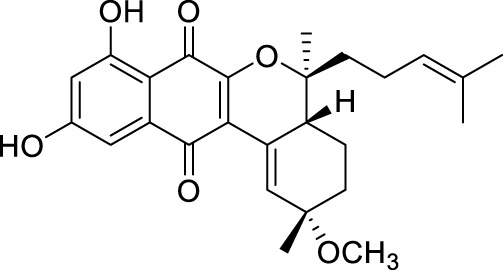	438.5	Hardt et al. (2000)
Neomarinone	C_26_H_32_O_5_	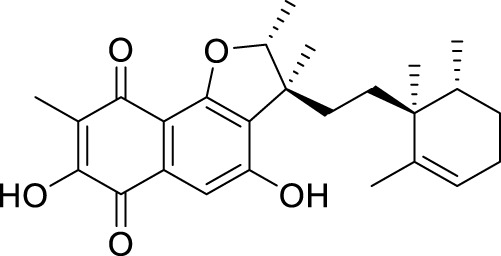	424.5	Hardt et al. (2000), Kalaitzis et al. (2003)

**FIGURE 1:**
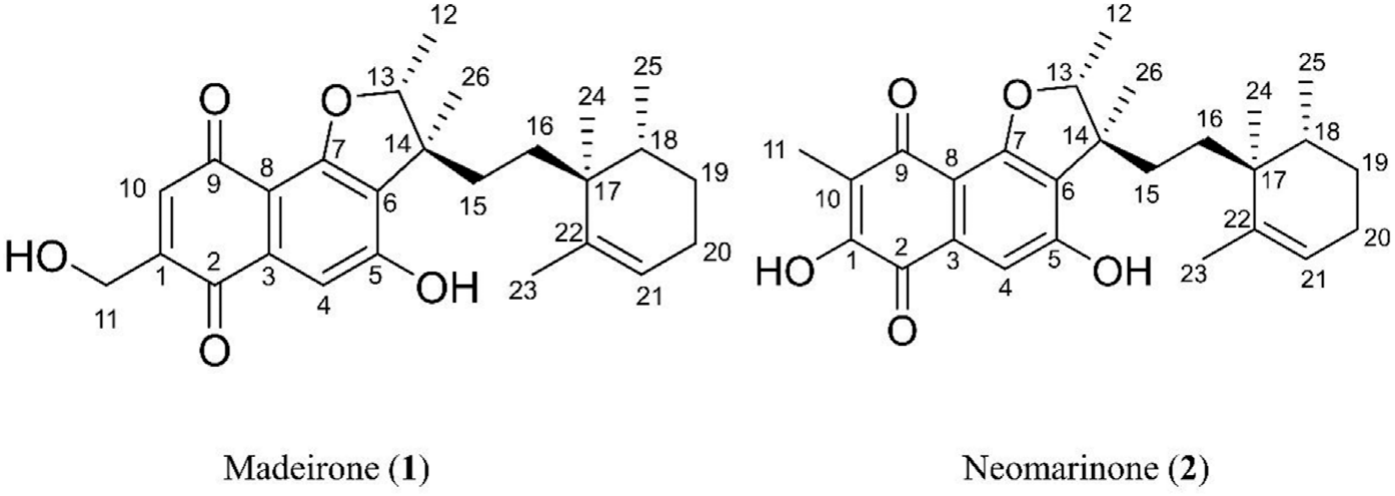
Chemical structures of madeirone (**1**) and neomarinone (**2**), isolated from marine-derived *S. aculeolatus* strain PTM-346.

The elucidation of compound (**1**) revealed a previously undescribed marinone derivative. This discovery expands the known class of marinones to seven, as shown in Table 2 and Figure 1. The novel compound (**1**) has been named madeirone, after the Madeira Archipelago, the origin of strain PTM-346.

**TABLE 2:** Obtained NMR data for the novel compound madeirone (**1**) in CDCl_3_. Protons were assigned via HSQC to the corresponding carbon atoms. ^1^H-spectra were recorded at 400 MHz,^13^C-NMR spectra at 100 MHz.

C/H	δ_C_ (ppm)	Carbon environment	δ_H_ (ppm)	Multiplicity, *J*, number of H-atoms	COSY (correlating δ_H_)	HMBC (correlating δ_C_)	NOESY (correlating δ_H_)
1	159.3	C=C					
2	180.1	C=O					
3	132.8	C=C					
4	109.5	C=C	7.14	s, 1H		2, 3, 5, 6, 8	
5	156.8	O-C=C					
6	128.9	C=C					
7	161.0	O-C=C					
8	109.3	C=C					
9	183.8	C=O					
10	111.2	C=C	5.96	s, 1H	11	1, 2, 8, 9	11
11	56.3	C-C	3.76	s, 2H	10	1	10
12	15.3	C-C	1.37	d, 6.6 Hz, 3H	13	13, 14	13, 26
13	88.0	C-O (Ether)	4.78	q, 6.6 Hz, 1H	12	15, 26	12
14	46.7	C-C					
15	31.7	C-C	1.24–1.14	m, 2H	16	6, 13, 14	
16	25.6	C-C	1.95–1.78	m, 2H	15		
17	40.3	C-C					
18	33.3	C-C	1.68	m, 1H	24, 19, 25	17, 19, 24, 25	25
19	27.1	C-C	1.32	m, 2H	18	17, 18, 21	25
20	31.1	C-C	1.95–1.78	m, 2H	21		21
21	124.6	C=C	5.33	s, 1H	20, 23		20, 23
22	139.1	C=C					
23	19.1	C-C	1.46	s, 3H	21	17, 21, 22	21, 24
24	21.2	C-C	0.72	s, 3H		17, 18, 22	23, 25, 26
25	15.9	C-C	0.74	m, 3H	18	18, 19	18, 19, 24
26	20.0	C-C	1.24–1.14	s, 3H		6, 13, 14, 15	12, 24

The identification of (**1**) was aided by MS analysis, which revealed a molecular mass of 424.2 Da, corresponding to the molecular formula C_26_H_32_O_5_. The molecular formula was further confirmed by the hydrogen and carbon atom counts obtained from the ^1^H- and ^13^C-NMR spectra. IR spectroscopic data revealed absorption bands at 3273 and 1,633 cm^−1^, characteristic of marinone and its derivatives (Hardt et al., 2000). Compound (1) shares the same molecular mass and structural formula as neomarinone (Table 1), a known compound with previously described antibiotic activity and cytotoxicity against human colon carcinoma cells HCT-116 (Pathirana et al., 1992; Hardt et al., 2000; Peña-López et al., 2009). However, analysis of the 1D- and 2D-NMR spectra revealed that compound (**1**) is a derivative and molecular isomer of neomarinone.

Further evidence of the novelty of compound (**1**) was provided through the elucidation of compound (**2**) as neomarinone. Identification of compound (**2**) was facilitated by HR-MS, which revealed a molecular mass of 424.225 Da, corresponding to the molecular formula of C_26_H_32_O_5_. The spectrometric and spectroscopic data obtained for compound (**2**) confirmed its identity as neomarinone (Table 3; Figure 1) (Hardt et al., 2000; Kalaitzis et al., 2003). The distinct structures of compounds (**1**) and (**2**) were further supported by their differing retention times in analytical HPLC analysis, with compound (1) having a retention time of 20.9 min and compound (2) having a retention time of 22.0 min (data not shown).

**TABLE 3:** Obtained NMR data for neomarinone (**2**) in DMSO-d6. Protons were assigned via HSQC to the corresponding carbon atoms. ^1^H-spectra were recorded at 400 MHz,^13^C-NMR spectra at 100 MHz.

C/H	δ_C_ (ppm)	Carbon environment	δ_H_ (ppm)	Multiplicity, *J*, number of H-atoms	COSY (correlating δ_H_)	HMBC (correlating δ_C_)	NOESY (correlating δ_H_)
1	152.6	O-C=C					
2	181.1	C=O					
3	130.6	C=C					
4	107.9	C=C	7.03	s, 1H		2, 3, 8	
5	157.2	O-C=C					
6	127.1	C=C					
7	159.7	O-C=C					
8	107.6	C=C					
9	183.2	C=O					
10	120.0	C=C					
11	7.6	C-C	1.98	s, 3H		1, 9, 10	
12	14.4	C-C	1.37	d, 6.6 Hz, 3H	13	13, 14	13, 26
13	86.1	C-O (Ether)	4.79	q, 6.6 Hz, 1H	12	15, 26	12
14	46.5	C-C					
15	30.5	C-C	1.19	m, 2H	16	6, 13, 14	
16	24.7	C-C	1.93–1.82	m, 2H	15		
17	40.3	C-C					
18	32.2	C-C	1.67	m, 1H	24, 19	25	25
19	26.1	C-C	1.34	m, 2H	18, 20		25
20	30.0	C-C	1.93–1.82	m, 2H	21, 19		21
21	123.3	C=C	5.34	s, 1H	20, 23		20, 23
22	138.2	C=C					
23	18.2	C-C	1.47	m, 3H	21	21, 22	21, 24
24	20.3	C-C	0.73	s, 3H		17, 18, 22	23, 25, 26
25	15.1	C-C	0.75	m, 3H	18	17, 18, 19	18, 19, 24
26	19.0	C-C	1.19	m, 3H		6, 13, 14, 15	12, 24

The HSQC experiment for compound (**1**) enabled the assignment of all proton signals to their corresponding carbon atoms. A comparative analysis of the 1D- and 2D-NMR spectra (Table 2; Table 3) for compounds (**1**) and (**2**) revealed that most of the chemical shifts and coupling patterns were identical. The structural differences of compound (**1**) compared to compound (**2**) were localized to the 1,4-benzoquinone moiety, specifically at C-1, C10 and C-11. Minor chemical shift variations were attributed to the use of different NMR solvents (CDCl_3_ for compound (**1**) and DMSO-d6 for compound (**2**)).

HSQC data for compound (**1**) revealed the assignment of one proton to C-10 and two protons to C-11, with no proton assigned to C-1. HMBC spectrum for compound (**1**) showed correlations of H-11 to C-1, and H-10 to C-1, C-2, C-8 and C-9. Additionally, COSY data showed coupling of H-10 with H-11. The ^1^H-NMR data depicted both H-10 and H-11 as singlets, indicating that these protons are not located on neighboring C-atoms.

The stereochemistry of compounds (**1**) and (**2**) was determined via NOESY data. For both compounds, strong correlations were observed between the chiral methyl groups H-12 and H-26, H-24 and H-25, as well as H-24 and H-26. The correlations indicate cis orientations of these groups, as previously reported for compound (**2**) in the literature (Hardt et al., 2000; Kalaitzis et al., 2003). Thus, the chirality of compounds (**1**) and (**2**) was found to be identical. Consequently, the combined NMR data of compound (**1**) suggests the substituted 1,4-benzoquinone moiety presented in Figure 1.

Notably, *S*. *aculeolatus* PTM-346 exhibits a significant distinction, regarding secondary metabolite profile. Since the other five strains of the same species obtained from the same location, the Madeira Archipelago, Portugal, produced a different class of meroterpenoid compounds termed napyradiomycins (Gaudêncio et al., 2016; Bauermeister et al., 2019; Pereira et al., 2020).

### 3.2 Marinones micro and macrofouling inhibitory activity assessment

The antimicrofouling activity of marinones derivatives (**1**) and (**2**) was assessed by examining their inhibitory effects on bacterial growth and on the formation of bacterial biofilms. For the bioactivity assays, five species of marine bacteria were selected, based on their proficiency in biofilm production and recognized as influential contributors to fouling, serving as primary colonizers on submerged surfaces (Dang et al., 2008). These bacterial models include, *P*. *inhibens* (DSM 17395), *P*. *batsensis* (DSM 15984), *M*. *hydrocarbonoclasticus* (DSM 8798), *C*. *marina* (DSM 4741) and *M*. *luteus* (DSM 20030, ATCC 4698) (El-Masry et al., 1995; Ekblad et al., 2008; Akesso et al., 2009; Briand, 2009; D’Souza et al., 2010; Inbakandan et al., 2010; Michael et al., 2016; Majzoub et al., 2018).

The antimacrofouling activity of marinone derivatives (**1**) and (**2**) was assessed concerning the settlement of plantigrade larvae of *M*. *galloprovincialis*.

#### 3.2.1 Antibacterial activity evaluation

The most promising compounds for antimicrofouling are those that inhibit biofilm formation without affecting the growth of the bacteria. The Minimum Biofilm Inhibitory Concentration (MBIC) is the value that inhibits biofilm formation by over 80% (upper threshold) and simultaneously inhibits bacterial growth by less than 40% (lower threshold) (Kwasny and Opperman, 2010; Bauermeister et al., 2019; Sabotič et al., 2024). To evaluate the anti-biofilm activity of the compounds, we first determined their antibacterial activity (Table 4).

**TABLE 4:** Percentage of growth inhibition for several marine bacteria in the presence of different concentrations of madeirone (**1**) and neomarinone (**2**). Shown are the average values of the percentage of growth inhibition of three replicates with the standard error of the mean (SEM). N.I—not inhibited. Results were statistically significant (****p < 0.0001, ***p < 0.001, **p < 0.01, *p < 0.05, Dunnet’s test).

		Growth inhibition (%)				
		Strain				
Compound	Tested conc [µg/mL]	*P. inhibens*	*P. batsensis*	*M. hydrocar bonoclasticus*	*C. marina*	*M. luteus*
Madeirone (**1**)	31.25	24.3 ± 0.5^****^	46.9 ± 1.6^****^	N.I.	12.7 ± 2.7^ns^	20.0 ± 0.5^****^
	15.60	N.I.	45.7 ± 0.4^****^	N.I.	7.7 ± 4.0	N.I.
	7.81	N.I.	35.9 ± 0.5^****^	N.I.	10.0 ± 2.1	N.I.
	3.91	N.I.	31.0 ± 0.7^****^	N.I.	N.I.	N.I.
	1.95	N.I.	26.6 ± 0.8^****^	N.I.	N.I.	N.I.
	0.98	N.I.	20.7 ± 1.1^****^	N.I.	N.I.	N.I.
Neomarinone (**2**)	31.25	N.I.	11.1 ± 0.4^****^	4.2 ± 0.8^*^	1.1 ± 2.0 ^ns^	N.I.
	15.60	N.I.	0.9 ± 0.4 ^ns^	N.I.	7.2 ± 2.9 ^ns^	N.I.
	7.81	N.I.	0 ± 0.3 ^ns^	N.I.	9.2 ± 2.1^*^	6.8 ± 1.0^*^
	3.91	N.I.	4.2 ± 0.3^****^	N.I.	6.5 ± 1.2 ^ns^	9.0 ± 0.9^**^
	1.95	N.I.	0.3 ± 0.5 ^ns^	N.I.	10.8 ± 3.1^*^	3.8 ± 1.7 ^ns^
	0.98	N.I.	N.I.	N.I.	10.7 ± 2.2^*^	1.8 ± 0.4 ^ns^
CuSO_4_	0.16	66.2 ± 5.6^****^	44.8 ± 6.3^****^	35.8 ± 0.8^****^	N.I.	N.I.
DMSO	250	N.I.	N.I.	N.I.	N.I.	N.I.

Madeirone (**1**) demonstrated a 24.3% ± 0.5% inhibition of *P. inhibens* growth for a concentration of 31.25 μg/mL and no inhibition for all the lower tested concentrations. It exhibited consistent inhibitory effects on *P. batsensis* across all tested concentrations, with inhibition values over 40% for concentrations 31.25 μg/mL and 15.60 μg/mL. No evident inhibition was observed for *M. hydrocarbonoclasticus* for any of the tested concentrations. Regarding the growth of *C. marina*, the compound showed inhibition percentages ranging from 12.7% ± 2.7% to 7.7% ± 4.0% at concentrations between 31.25 and 7.81 μg/mL. *M. luteus* growth was inhibited by 20.0% ± 0.5% and 9.2 %± 0.2% at a concentration of 31.25 μg/mL and 15.60 μg/mL, respectively.

Neomarinone (**2**) did not inhibit the growth of *P. inhibens* at any of the tested concentrations. It exhibited no to low inhibitory effects on *P. batsensis*, at concentrations ranging from 31.25 to 1.95 μg/mL, resulting in inhibition percentages ranging from 0.0% ± 0.3% to 11.1% ± 0.4% at the highest concentration. The growth of *M. hydrocarbonoclasticus* was only inhibited by 4.2% ± 0.8% at 31.25 μg/mL. *C. marina* was inhibited at tested concentrations from 1.1% ± 2.0% to 10.7% ± 2.2%, while compound (**2**) inhibited *M. luteus* at concentrations from 7.81 to 0.98 μg/mL, resulting in inhibition percentages ranging from 9.0% ± 0.9% to 1.8% ± 0.4%.

CuSO_4_, a highly effective antifouling agent utilized in antifouling paints, served as the reference in this study, at a concentration of 0.16 μg/mL. The application of CuSO_4_ resulted in significant inhibitions in the growth of *P. inhibens*, *P. batsensis*, and *M. hydrocarbonoclasticus*, with inhibitory percentages of 66.2% ± 5.6%, 44.8% ± 6.3%, and 35.8% ± 0.8%, respectively. No inhibition of growth was observed for *M. luteus* and *C. marina* under the influence of CuSO_4_. In DMSO no bacterial growth inhibition was observed for any of the tested strains in the presence of both madeirone (**1**) and neomarinone (**2**).

The results of growth inhibition of marine bacteria obtained for the metabolite madeirone (**1**) were <25% at the highest tested concentration for almost all tested bacteria (*P*. *inhibens, M. hydrocarbonoclasticus*, *M. luteus* and *C. marina*), except for *P. batsensis* where the growth inhibition was close to 50%. The growth inhibition for neomarinone (**2**) were around 10% or lower for *P. batsensis*, *C. marina*, *M. hydrocarbonoclasticus*, and *M. luteus* at the highest tested concentration.

Thus, both compounds showed growth inhibition activity mostly below the 40% threshold considered for antifouling activity (Kwasny and Opperman, 2010; Bauermeister et al., 2019; Sabotič et al., 2024), and below the values obtained for CuSO_4_, emerging as promising compounds for marine intervention, since they do not have a killing effect on marine bacteria.

#### 3.2.2 Antibiofilm activity assessment

Madeirone (**1**) effectively hindered the biofilm formation of *P. batsensis* and *C. marina* across all tested concentrations, exhibiting inhibitory percentages ranging from 10.8% ± 1.7% to 59.2% ± 0.4%. The biofilm formation of *P. inhibens* was impeded at concentrations ranging from 31.25 μg/mL to 3.91 μg/mL, resulting in inhibition percentages ranging from 66.7% ± 0.3% to 10.2% ± 3.7%, respectively. Similarly, *M. hydrocarbonoclasticus* biofilm formation showed inhibition at concentrations ranging from 7.81 μg/mL to 31.25 μg/mL, with inhibitory percentages ranging from 14.2% ± 3.6% to 60.1% ± 0.5%, respectively. *M*. *luteus* reveals inhibition of 35.3% ± 0.2% only at the highest tested concentration of 31.25 μg/mL (Table 5).

**TABLE 5:** Percentage of inhibition of biofilm formation for several marine bacteria in the presence of different concentrations of madeirone (**1**) and neomarinone (**2**). Shown are the average values of the percentage of biofilm inhibition of three replicates with the standard error of the mean (SEM). N.I—not inhibited. Results were statistically significant (****p < 0.0001, ***p < 0.001, **p < 0.01, *p < 0.05, Dunnet’s test).

		Biofilm inhibition (%)				
		Strain				
Compound	Tested conc [µg/mL]	*P. inhibens*	*P. batsensis*	*M. hydrocar bonoclasticus*	*C. marina*	*M. luteus*
Madeironae (**1**)	31.25	66.7 ± 0.3^****^	59.2 ± 0.4^****^	60.1 ± 0.5^****^	31.0 ± 9.6 ^ns^	35.3 ± 0.2^****^
	15.60	53.0 ± 1.0^****^	49.1 ± 0.7^****^	33.9 ± 2.1^*^	41.7 ± 10.0 ^ns^	N.I.
	7.81	33.9 ± 1.3^****^	34.8 ± 2.7^****^	14.2 ± 3.6^*^	39.2 ± 5.4 ^ns^	N.I.
	3.91	10.2 ± 3.7 ^ns^	24.9 ± 2.9^****^	N.I.	36.3 ± 6.4 ^ns^	N.I.
	1.95	N.I.	17.9 ± 2.4^****^	N.I.	31.6 ± 15.3 ^ns^	N.I.
	0.98	N.I.	10.8 ± 1.7^**^	N.I.	28.6 ± 23.2 ^ns^	N.I.
Neomarinone (**2**)	31.25	41.3 ± 0.9^****^	40.7 ± 4.7^***^	29.0 ± 10.1^**^	45.8 ± 2.9^****^	33.3 ± 7.3 ^ns^
	15.60	30.0 ± 0.6^**^	34.9 ± 4.8^***^	56.7 ± 1.5^****^	42.6 ± 6.3^***^	41.0 ± 8.2 ^ns^
	7.81	11.6 ± 1.8^**^	26.3 ± 5.9^**^	54.4 ± 1.8^****^	34.4 ± 7.4^**^	29.9 ± 12.4 ^ns^
	3.91	N.I.	19.4 ± 4.0 ^ns^	54.5 ± 3.6^****^	26.9 ± 3.3^**^	7.8 ± 16.9 ^ns^
	1.95	N.I.	13.9 ± 5.7 ^ns^	58.1 ± 1.6^****^	24.5 ± 3.0^**^	N.I.
	0.98	N.I.	11.7 ± 6.2 ^ns^	49.2 ± 6.2^****^	26.5 ± 3.8^**^	N.I.
CuSO_4_	0.16	12.9 ± 6.4	41.4 ± 0.9	N.I.	N.I.	N.I.
DMSO	250	N.I.	N.I.	N.I.	N.I.	N.I.

In contrast, neomarinone (**2**) exhibited inhibitory effects on the biofilm formation *of P. batsensis*, *M. hydrocarbonoclasticus*, and *C. marina* at all tested concentrations, with the lowest inhibition at 11.7% ± 6.2% and the highest at 58.1% ± 1.6%. The biofilm formation of *P. inhibens* was hindered at concentrations ranging from 7.81 to 31.25 μg/mL, resulting in inhibition percentages ranging from 11.6% ± 1.8% to 41.3% ± 0.9%, respectively. Moreover, *M. luteus* inhibition occurred at concentrations ranging from 3.91 to 31.25 μg/mL, with inhibitory percentages ranging from 7.8% ± 16.9% to 41.0% ± 8.2% (Table 5).

Madeirone (**1**) showed biofilm inhibition results exceeding 50% for *P. inhibens* at 15.6 μg/mL, and *M. hydrocarbonoclasticus at* 31.35 μg/mL, and surpassing 30% for *C. marina* at all tested concentrations. At these concentration values, the growth inhibition was below 10%. The only exception was *P. batsensis,* for which the growth inhibition showed similar values than the ones for biofilm inhibition.

Neomarinone (**2**) demonstrated more than 40% inhibition of biofilm formation in all tested marine fouling bacteria. Specifically, at a concentration of 31.35 μg/mL, it exhibited inhibition for *P. inhibens* and *P. batsensis*, at 15.60 μg/mL for *M. luteus*, and at 15.60 and 31.25 μg/mL for *C. marina*. Notably, it displayed inhibition ranging from 40% to 60% for *M. hydrocarbonoclasticus* at all concentrations except 31.25 μg/mL. For neomarinone (**2**), the values of growth inhibition were below 10% or only slightly over this value.

CuSO_4_ only inhibited the biofilm formation of *P. batsensis*, with an inhibitory percentage of 41.4% ± 0.9%. There was no observable inhibition in the biofilm formation of *M. luteus*, *M. hydrocarbonoclasticus* and *C. marina*, and for *P*. *inhibens* the inhibition was only 12.9% ± 6.4%. No inhibition of biofilm formation was observed in DMSO for any of the tested strains in the presence of both madeirone (**1**) and neomarinone (**2**).

Although the values of percentage of biofilm inhibition were lower (between ∼40 and 66%) than the 80% threshold usually used to establish the MBIC value (Kwasny and Opperman, 2010; Bauermeister et al., 2019; Sabotič et al., 2024), we considered that the two compounds in study have promising antibiofilm activity, due to their very low effect on growth inhibition (0–∼20%).

Overall, madeirone (**1**) exhibited promising antibiofilm efficacy against *P. inhibens*, up to 66% inhibition, *M. hydrocarbonoclasticus* (up to 60% inhibition) and *C. marina* (up to 40% inhibition), along with growth inhibition activity below 10%, for the same respective concentrations of this compound.

Neomarinone (**2**) also displayed positive antibiofilm outcomes, with up to 41% inhibition against *P. inhibens*, 40% inhibition against *P. batsensis*, 56% inhibition against *M. hydrocarbonoclasticus,* 46% inhibition against *C. marina*, 40% inhibition against *M. luteus*, along with growth inhibition activity below 10%, for the same respective concentrations of this compound.

It is noteworthy that, except for madeirone (**1**) against *P. batsensis*, negligible growth inhibition was observed. This emphasizes the compound’s effectiveness as potent antibiofilm agents without compromising the viability of the targeted bacteria, a crucial point that is reinforced by this study. The antibiofilm activity of these marinone derivatives is independent of their antibacterial effects. This suggests that these compounds could serve as antibiofilm agents without contributing to antibiotic/biocide resistance.

Several reported studies involving *Streptomyces* compounds have highlighted their antibiofilm activity by referencing the Minimum Inhibitory Concentration (MIC) for growth inhibition. However, it is essential to clarify that the focus should be on the Minimum Biofilm Inhibitory Concentration (MBIC), which denotes the inhibition of biofilm growth without adversely affecting bacterial growth (Selvin, 2009; Cho and Kim, 2012; Gopikrishnan et al., 2016; Kavitha and Vimala, 2020; She et al., 2022; Stalin et al., 2022).

Our group previously reported the antifouling activity of napyradiomycins derivatives, meroterpenoides isolated from *S*. *aculeolatus* obtained from ocean sediments collected in the Madeira Archipelago. Napyradiomycins inhibited ≥80% of the marine biofilm formation for the same assayed bacteria. In comparison, marinones revealed lower antibiofilm inhibition than napyradiomycins, but also lower growth inhibition rates (Pereira et al., 2020). In comparison to napiradionycins, marinones hold a distinct edge as they inhibit antibiofilm-forming bacteria more effectively, and at lower concentrations. Both marinones and napyradiomycins surpass existing commercial biocides due to their non-toxic nature and their ability to inhibit microfouling.

#### 3.2.3 Assessment of antifouling properties on *Mytilus galloprovincialis* larval settlement

The antimacrofouling activities of compounds (**1**) and (**2**) were assessed against the plantigrade larval settlement of *M. galloprovincialis* and exhibited EC_50_ values of 1.755 and 0.119 μg/mL, respectively (Table 6).

**TABLE 6:** Settlement response of *M. galloprovincialis* plantigrade larvae to marinone derivatives (**1**, **2**) after a 15 h acute exposure assay. The therapeutic ratio (LC_50_/EC_50_) was used to assess the efficacy of each compound relative to its toxicity. Negative control: DMSO = 100% settlement; Positive control: 0.16 μg/mL CuSO_4_ = 0% settlement.

Compound	EC_50_ [Conf. Limits] (µg/mL)	LC_50_ (µg/mL)	LC_50_/EC_50_
(**1**)	1.755 (1.308–5.492)	>10	5.80
(**2**)	0.119 (0.015–0.253)	>4	35.71

The marinones used in this study demonstrated notable effectiveness, with an EC_50_ value much lower than the advisable threshold for compounds consideration as effective AF agents, 25 μg/mL (Almeida and Vasconcelos, 2015). Remarkably, the EC_50_ of (**2**) was lower than that of ivermectin (EC_50_ = 0.4 μg/mL against *M. edulis*) (Davies et al., 1997) and lower than other promising reported compounds isolated from *Streptomyces* (Selvin, 2009; Cho, 2012; Cho et al., 2012; Cho and Kim, 2012; Prakash et al., 2015; Gopikrishnan et al., 2016), similar to the most promising napyradiomycins previously reported by our group from actinomycetes *S*. *aculeolatus*. This napyradiomycins displayed settlement results of *M*. *galloprovincialis* larvae with an EC_50_ of less than 5 μg/mL and a LC_50_/EC_50_ ratio greater than 15 (Pereira et al., 2020). Further, the EC_50_ of (**2**) was lower than that of other synthesized compounds or compounds from distinct bioresources (Neves et al., 2021; Pereira et al., 2021; Resende et al., 2021).

Regarding toxicity, none of the tested marinone derivatives caused mortality to *M. galloprovincialis* larvae at the maximum tested dose (10 μg/mL). Consequently, LC_50_ values exceeding 10 and 4 μg/mL, respectively were considered, and therapeutic ratios (LC_50_/EC_50_) were computed using the EC_50_ and LC_50_ values. To meet the standard requirement for the efficacy of natural antifouling agents, the US Navy program established (LC_50_/EC_50_) > 15 as a therapeutic ratio cut-off (Qian et al., 2009). Consequently, particularly neomarinone (**2**) (LC_50_/EC_50_ = 35.71) emerges as the most promising antifouling marinone-derivative agent for *M. galloprovincialis larvae* (Table 6).

### 3.3 *In silico* ecotoxicity analysis

The growing concern regarding the ecotoxicity of various pharmaceuticals, biocides, and chemical compounds has prompted regulatory authorities to advocate for the adoption of *in silico* risk assessment methodologies. Employing the Toxicity Estimation Software Tool (T.E.S.T.) (CCTE, EPA 2022), compounds (**1**) and (**2**) underwent a comprehensive evaluation to assess their potential ecotoxicity. For a comparative analysis, approved drugs such as paracetamol (**3**) and methicillin (**4**) were included, along with antifouling agents lobocompactol (**5**), invermictin B1b (**6**), and B1a (**7**) (commercialized ivermectin is a mixture of two molecular derivatives: 80% ivermectin B1a with an ethyl group at position C-26% and 20% ivermectin B1b with a methyl group at position C-26 (Pinori et al., 2011)), copper (**8**), and arsenic (**9**) (Table 7, 8). These approaches facilitate the anticipation of the fate of these molecules, their potential ecological impact, and potential indirect effects on human health.

**TABLE 7:** Madeirone (**1**), neomarinone (**2**) and approved drugs (**3**-**7**) predicted toxicity endpoints.

Toxicity end points for Consensus models							
No.	Fathead minnow ^a^	*Daphnia magna* ^b^	*Tetrahymena pyriformis* ^c^	Oral rat ^d^	Bioconcentration factor	Developmental toxicity ^e^	Ames mutagenicity ^f^
**1**	0.02	0.73	0.20	61.10	45.50	0.98; DT	0.07; MN
**2**	0.02	0.76	4.67	84.73	50.66	0.98; DT	−0.05; MN
**3**	89.69	27.14	273.83	1684.75	1.73	0.46 DNT	0.45; MN
**4**	0.90	67.23	4.19	4097.93	1.23	0.03 DNT	−0.01; MN
**5**	0.64	2.67	5.54	122.13	66.62	0.66 DT	0.14; MN
**6**	0.05	12.91	10.77	29.69	1.99	0.44; DNT	0.13; MN
**7**	0.002	15.81	75.78	30.31	2.20	0.50; DNT	0.25; MN

^a^ 96 h LC_50_ (mg/L).

^b^ 48 h LC_50_ (mg/L).

^c^ 48 h IGC_50_ (mg/L), the Nearest Neighbour model, the other models are unable to predict this end point.

^d^ LD_50_ (mg/kg).

^e^ DT: developmental toxicant, DNT: Developmental Non-Toxicant, DT: Developmental Toxicant.

^f^ MN: mutagenicity negative.

**TABLE 8:** Aquatic toxicity, environmental fate data and classification of copper and arsenic (Tišler and Zagorc-končan, 2003).

	Toxicity end points					
No.	Fish *Orcorhynchus mykiss* ^a^	*Daphnia magna* ^b^	Alga *Scenedesmus quadricauda* ^d^	*Daphnia magna* ^d^	Oral rat/rabit^e^	Bioconcentration Factor
**8**	0.48	0.030	0.18	0.015	140	Irrelevant
**9**	15.3	2.5	34.5	1.85	12	Irrelevant

^a^ 96 h LC_50_ (mg/L).

^b^ 48 h LC_50_ (mg/L).

^c^ 48 h IGC_50_ (mg/L).

^d^ 21 days NOEC (mg/L).

^e^ LD_50_ (mg/kg).

The Toxicity Estimation Software Tool (T.E.S.T.) does not predict the toxicity of salts, ions, or metals. Thus, for copper and arsenic the reported data of Tisler and Zagorc-Koncan is used (Tišler and Zagorc-končan, 2003) (Table 8).

In accordance with the European Union Directive 2001/59/EC and Regulation 1,272/2008 on the Classification, Labelling, and Packaging of Substances and Mixtures (CLP), the classification of a substance as “harmful,” “toxic,” or “very toxic” to aquatic organisms is determined based on various criteria. These criteria include the 96-h LC50 for fish (e.g., fathead minnow), 48 h LC_50_ for daphnids (e.g., *D. magna*), and other assays like the 72 h IC_50_ for algae or 40 h IGC_50_ for protozoans (e.g., *Tetrahymena pyriformis*). If the IC_50_, LC_50_, or IGC_50_ falls below 1 mg/L, the substance is labeled as “very toxic to aquatic organisms” (indicated by the danger symbol N and risk phrase R50). Values between 1 and 10 mg/L classify the substance as “toxic to aquatic organisms” (danger symbol N, risk phrase R51), while endpoints between 10 and 100 mg/L result in classification as “harmful to aquatic organisms” (risk phrase R52). Additionally, classification considers factors such as ready biodegradability and bioaccumulation potential, assessed through the bioconcentration factor (BCF). If the BCF is ≥ 100, the compound is categorized as “may cause long-term adverse effects in the aquatic environment” (risk phrase R53) (Tišler and Zagorc-končan, 2003; Schipper et al., 2010). Acute Toxicity Estimates (ATE) categories, as outlined by the CLP regulation, depend on the Oral rat LD_50_ (Diaza et al., 2015). The four ATE thresholds are as follows: 1) category 1, ATE ≤5 mg/kg, designating the substance as “Fatal if swallowed”; 2) category 2, 5 < ATE ≤50 mg/kg, also classified as “Fatal if swallowed”; 3) category 3, 50 < ATE ≤300 mg/kg, labeled as (“Toxic if swallowed”); 4) category 4, 300 < ATE ≤2000 mg/kg, categorized as “Harmful if swallowed”; and 5) category 5, ATE >2000 mg/kg, indicating that the substance “may be Harmful if swallowed”. For the evaluation of toxicity toward humans, mutagenicity, carcinogenicity, and reproductive toxicity are crucial endpoints. Mutagenic toxicity can be experimentally assessed through various test systems, with the Ames test being the most common, which uses genetically engineered *Salmonella typhimurium and Escherichia coli* bacterial strains (Gini et al., 2014).

Analysis of the *in silico* results from the Toxicity Estimation Software Tool (T.E.S.T.) (table 7 and 8) enabled the categorization of the toxicity endpoints of compounds (**1**)–(**9**) (Table 9).

**TABLE 9:** Prediction of toxicity endpoint of madeirone (**1**), neomarinone (**2**) and approved drugs (**3**-**9**).

Toxicity estimates		
No.	R phrases, danger symbol	ATE category
(**1**)	N, R50	3
(**2**)	N, R50	3
(**3**)	R52	4
(**4**)	N, R50	5
(**5**)	N, R50	3
(**6**)	N, R50	3
(**7**)	N, R50	3
(**8**)	N, R50/53	3
(**9**)	N, R50/53	2

Madeirone (**1**) was assigned the danger symbol N (“dangerous for the environment”), risk phrase 50 (“very toxic to aquatic organisms”) and ATE 3 (“Toxic if swallowed”), neomarinone (**2**) was also categorized with symbol N (“dangerous for the environment”), risk phrase 50 (“very toxic to aquatic organisms”), but ATE 3 (“Toxic if swallowed”), meaning that compound (**2**) has higher Acute Toxicity Estimate category. Additionally, these marinone derivatives were identified as developmental toxicants with a low bioaccumulation factor and negative mutagenicity.

The approved drug paracetamol (**3**) has been classified as “harmful to aquatic organisms” (risk phrase R52) and ATE 4 (“Harmful if swallowed”). In contrast, the antibiotic methicillin (**4**) bears the danger symbol N (“dangerous for the environment”), risk phrase 50 (“very toxic to aquatic organisms”), and ATE 5 “may be Harmful if swallowed,” which does not differ significantly from the classifications of the studied marinone derivatives.

*In silico* values related to environmental toxicity for compounds (**1**, **2**) (Table 7) showed similar orders of magnitude and classifications to those of the selected antibiofouling agents lobocompactol (**5**), invermictin B1b (**6**), and B1a (**7**), which all share the classification of symbol N (“dangerous for the environment”), risk phrase 50 (“very toxic to aquatic organisms”), and ATE 3 “Toxic if swallowed".

Copper (**8**) and arsenic (**9**) are also categorized with the symbol N (“dangerous for the environment”) and risk phrase 50 (“very toxic to aquatic organisms”). However, copper (**8**) has an ATE 3 “Toxic if swallowed,” while arsenic (**9**) has a higher ATE 2 “Fatal if swallowed.” Moreover, their BCF is ≥ 100, leading to their categorization with risk phrase R53, indicating that they “may cause long-term adverse effects in the aquatic environment” (Table 9) (Tišler and Zagorc-končan, 2003).

Globally, the *in silico* results suggest that madeirone (**1**) and neomarinone (**2**) are promising models for testing against Naval Sea Systems Command (NAVSEA) standards and advancing in the development roadmap for antifouling coatings (http://www.nstcenter.biz/navy-product-approval-process/navy-community-coatings-roadmap/, accessed on 26 January 2024).

### 3.4 *In vivo* ecotoxicity analysis

Madeirone (**1**) and neomarinone (**2**) were evaluated for acute toxicity towards *A. fischeri* and *D. magna* and chronic toxicity against *P. subcapitata* and *P. tricornutum* (Table 10).

**TABLE 10:** Percentage of inhibition towards *Aliivibrio fischeri*, *Daphnia magna, Pseudokirchneriella subcapitata* and *Phaeodactylum tricornutum* at the tested concentrations of madeirone (**1**) and neomarinone (**2**). Shown are the average values of the percentage of three replicates. Only values with <10% SD are presented. N.I—not inhibited.

		Inhibition (%)			
Compound	Tested conc [µg/mL]	*A. fischeri*	*D. magna*	*P. subcapitata*	*P. tricornutum*
Madeirone (**1**)	100	49.2	NI	52.8	86.9
	50	15.1	NI	27.3	58.0
	25	N.I	NI	9.1	45.7
	12.5	N.I	NI	N.I	N.I.
	6.25	N.I	NI	N.I.	N.I.
	3.13	N.I	NI	N.I.	N.I.
	1.56	N.I	NI	N.I.	N.I.
Neomarinone (**2**)	100	37.3	33.0	26.2	77.7
	50	25.0	NI	9.7	40.8
	25	N.I	NI	N.I.	38.5
	12.5	N.I	NI	N.I.	26.5
	6.25	N.I	NI	N.I.	N.I.
	3.13	N.I	NI	N.I	N.I.
	1.56	N.I	NI	N.I.	N.I.
Phenol	100	19.8	─	─	─
K_2_Cr_2_O_7_	100	─	1.7	0.45	18.5
DMSO	100	NI	NI	NI	NI

Madeirone (**1**) inhibited 49.2% the bioluminescence of *A. fischeri* at 100 μg/mL the highest tested concentration and 15.1% at 50 μg/mL. Neomarinone (**2**) demonstrated a similar toxicity of 37.3% and 25.0% at 100 μg/mL and 50 μg/mL against *A. fischeri*. *D. magna* was not immobilized at any of the concentrations tested for madeirone (**1**) and was immobilized by 33% at the highest tested concentration (100 μg/mL) for neomarinone (**2**) (Table 10).

The chronic ecotoxicity tests using algae *P. subcapitata* and *P. tricornutum* demonstrated that madeirone (1) was not toxic at the lowest tested concentrations (<12.5 μg/mL) (Table 10). Neomarinone (2) revealed no toxicity at concentrations ≤25 μg/mL against *P. subcapitata and at* concentrations ≤6.25 μg/mL *for P. tricornutum* (Table 10).

*In vivo* ecotoxicity studies highlight the compounds’ potential for further development as antifouling agents.

## 4 Conclusion

Novel metabolite madeirone (**1**) and neomarinone (**2**) exhibit a dual capability by effectively inhibiting both micro and macrofouling, distinguishing them from other commercial compounds and positioning them as agents for biofouling prevention. These possess antibiofilm and antifouling activities against species that contribute to fouling formation, without compromising their viability. These marinone derivatives not only impede the growth of various fouling organisms but also exhibit a minimal harmful impact on the marine ecosystem. Consequently, these compounds emerge as a promising alternative in the formulation of paints, varnishes, primers, and sealants, offering advantages over the use of copper compounds. In detail, madeirone (**1**) demonstrated significant antibiofilm efficacy against *P. inhibens*, achieving up to 66% inhibition, against *M. hydrocarbonoclasticus* (up to 60% inhibition) and *C. marina* (up to 40% inhibition). However, no biofilm inhibition was observed against *M. luteus* and similar antimicrobial and antibiofilm activity was observed against *P. batsensis*, with inhibition rates of up to 47% and 60%, respectively. Neomarinone (**2**) exhibited positive antibiofilm outcomes against all tested marine bacteria, with up to 41% inhibition against *P. inhibens*, 40% inhibition against *P. batsensis*, and 56% inhibition against *M. hydrocarbonoclasticus*. Furthermore, it demonstrated promising antibiofilm activity against *C. marina*, achieving up to 46% inhibition and against *M. luteus*, with 40% inhibition. In all cases, the growth inhibition activity was lower than 10%, for the same respective concentrations of the compounds. This underscores the compounds’ effectiveness as potent antibiofilm agents without compromising the viability of the targeted bacteria, a critical point that must be emphasized in this study. In fact, the antibiofilm activity of compounds (**1**) and (**2**) is independent of their antibacterial effects, suggesting that these could serve as antibiofilm agents without contributing to antibiotic/biocide resistance. Novel madeirone (**1**) and neomarinone (**2**), which was previously reported as having anticancer activity, are herein described as potent marine antibiofilm inhibitors and anti-macrofouling agents.

Madeirone (**1**) and neomarinone (**2**) also produced potent effects against the settlement of *M. galloprovincialis* larvae (EC_50_ = 1.76 and 0.12 μg/mL, respectively), along with no induced toxicity.

*In silico* environmental impact were predicted for madeirone (**1**) and neomarinone (**2**), with (**1**) assigned the danger symbol N, risk phrase 50, and ATE 4, while (**2**) shared the same symbol and risk phrase but had a higher ATE 3, indicating greater acute toxicity. These marinone derivatives were also identified as developmental toxicants with low bioaccumulation and as having negative mutagenicity. The compounds suggest not having higher toxicity than several approved drugs and antifouling agents and are less harmful than copper or arsenic. *In vivo* ecotoxicity studies emphasize the potential of compounds (**1**) and (**2**) as eco-friendly antibiofilm and antifouling agents.

## Data Availability

The datasets presented in this study can be found in online repositories. The names of the repository/repositories and accession number(s) can be found in the article/Supplementary Material.
