# Supplementary material for: Novel metabolite madeirone and neomarinone extracted from Streptomyces aculeoletus as marine antibiofilm and antifouling agents
Source: Front Chem. 2024 Jul 25;12:1425953. doi: 10.3389/fchem.2024.1425953 (PMC11306024; doi:10.3389/fchem.2024.1425953)
Supplement: Supplementary file 1 [file DataSheet1.docx]

Supplementary Material

Novel metabolite madeirone and neomarinone extracted from *Streptomyces aculeoletus* as marine antibiofilm and antifouling agents

Julian L. Wissner^1,2,3^, Joana R. Almeida^4^, Inês R. Grilo^1,2^, Jhenifer F. Oliveira^1,2^, Carolina Brízida^1,2^, Wendy Escobedo-Hinojosa^1.2.3^, Panayiota Pissaridou,^5^ Marlen I. Vasquez,^5^ Isabel Cunha^4^, Rita G. Sobral^1,2^, Vítor Vasconcelos^4,6^, Susana P. Gaudêncio^1,2*^

^1^ Associate Laboratory i4HB, Institute for Health and Bioeconomy, NOVA Faculty of Sciences and Technology, NOVA University of Lisbon, 2819-516 Lisbon, Portugal

^2^ UCIBIO, Applied Molecular Biosciences Unit, Chemistry and Life Sciences Departments, NOVA Faculty of Sciences and Technology, NOVA University of Lisbon, 2819-516 Lisbon, Portugal

^3^ Unidad de Química en Sisal, Facultad de Química, Universidad Nacional Autónoma de México, Puerto de abrigo s/n, 97356 Sisal, Yucatán, México

^4^ CIIMAR/CIMAR—Interdisciplinary Centre of Marine and Environmental Research, University of Porto, Terminal de Cruzeiros do Porto de Leixões, Avenida General Norton de Matos, 4450-208 Matosinhos, Portugal

^5^ Department of Chemical Engineering, Cyprus University of Technology, Archiepiskopou Kyprianou 30, 3036 Limassol, Cyprus

^6^ Biology Department, Faculty of Sciences, Porto University, Rua do Campo Alegre, 4069-007 Porto, Portugal

*** Correspondence:**Corresponding Author
s.gaudencio@fct.unl.pt


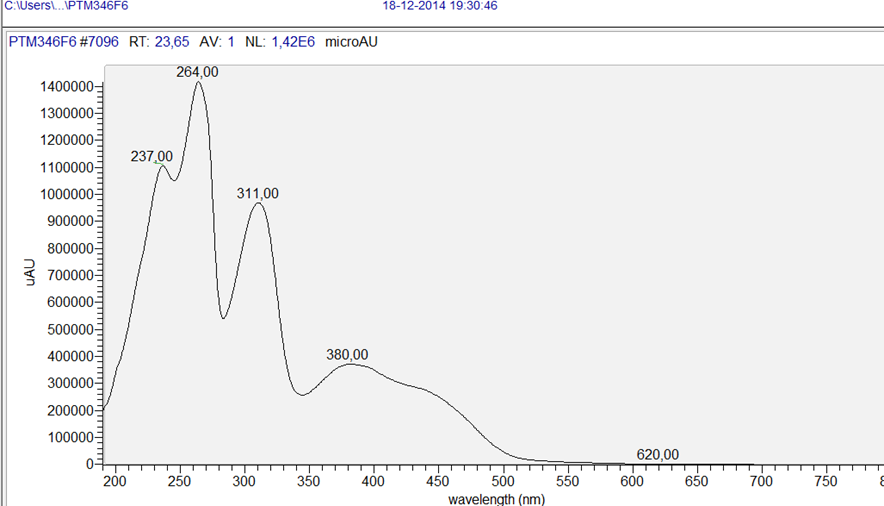


**Figure S1.** General UV spectrum profile of marinone class.


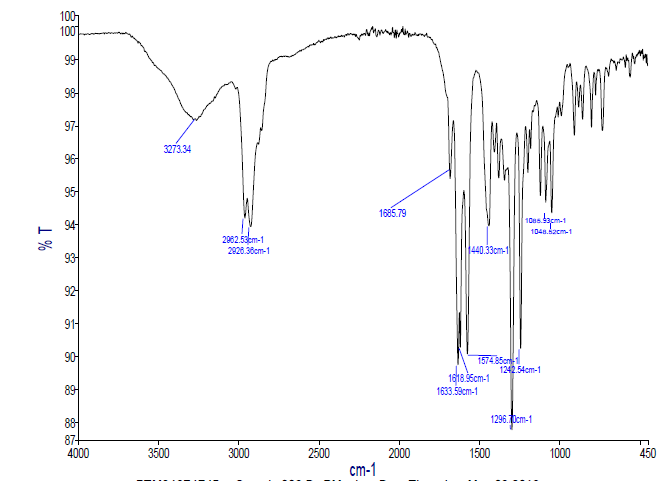


**Figure S2.** IR spectrum of madeirone (**1**)


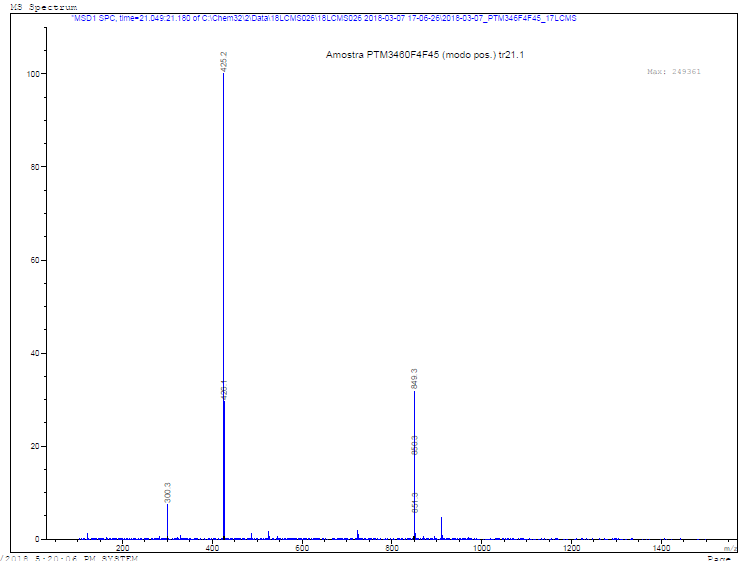


**Figure S3**. Mass spectrum (*m/z*) of madeirone (**1**) in positive mode. Fragmentation pattern: 425.2 [M+H]^+^, 849.3 [2M+H]^+^.


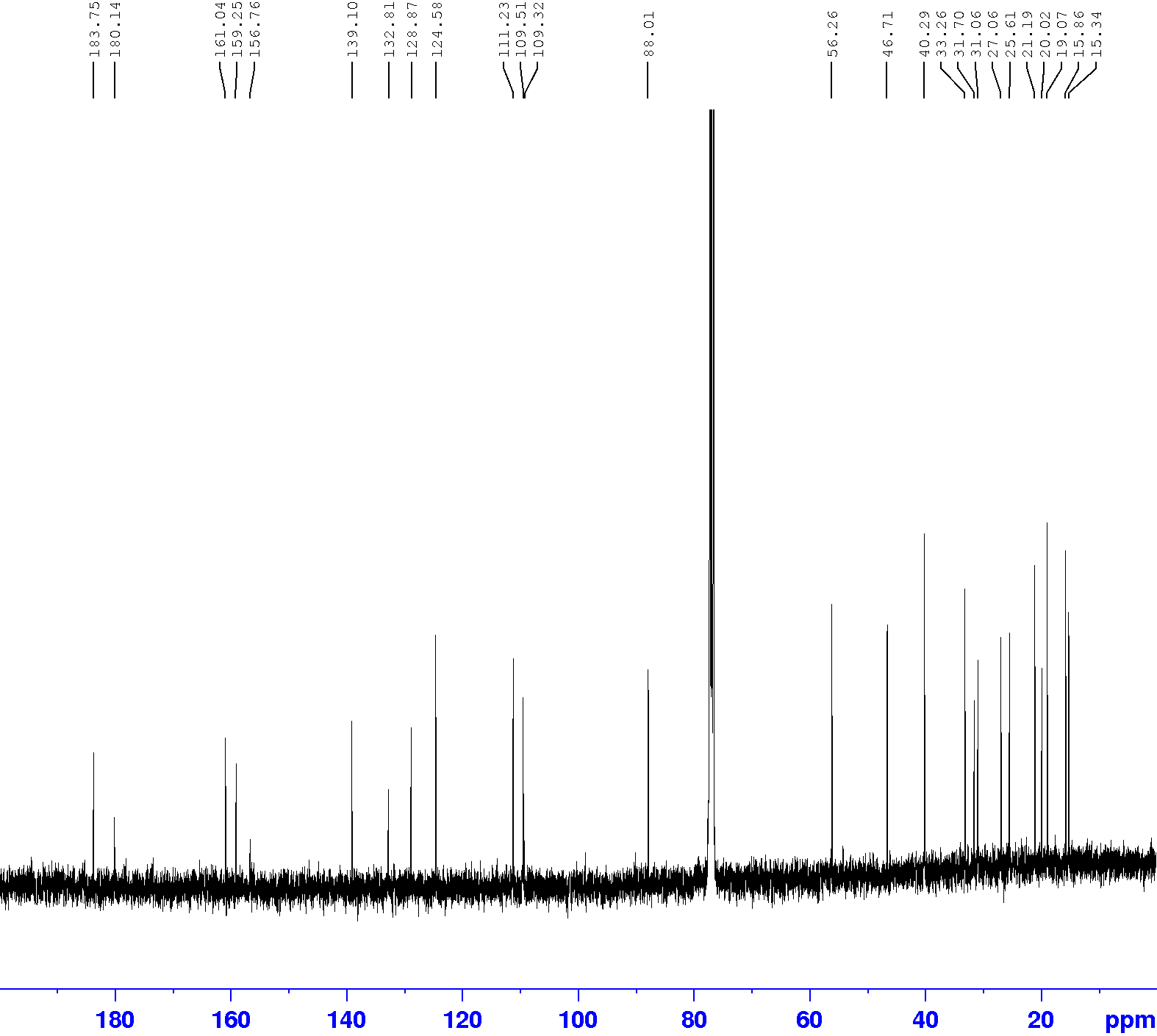


Figure S4. ^13^C-NMR spectrum of the novel compound madeirone (1), recorded at 100 MHz in CDCl_3_ with TMS as internal reference.


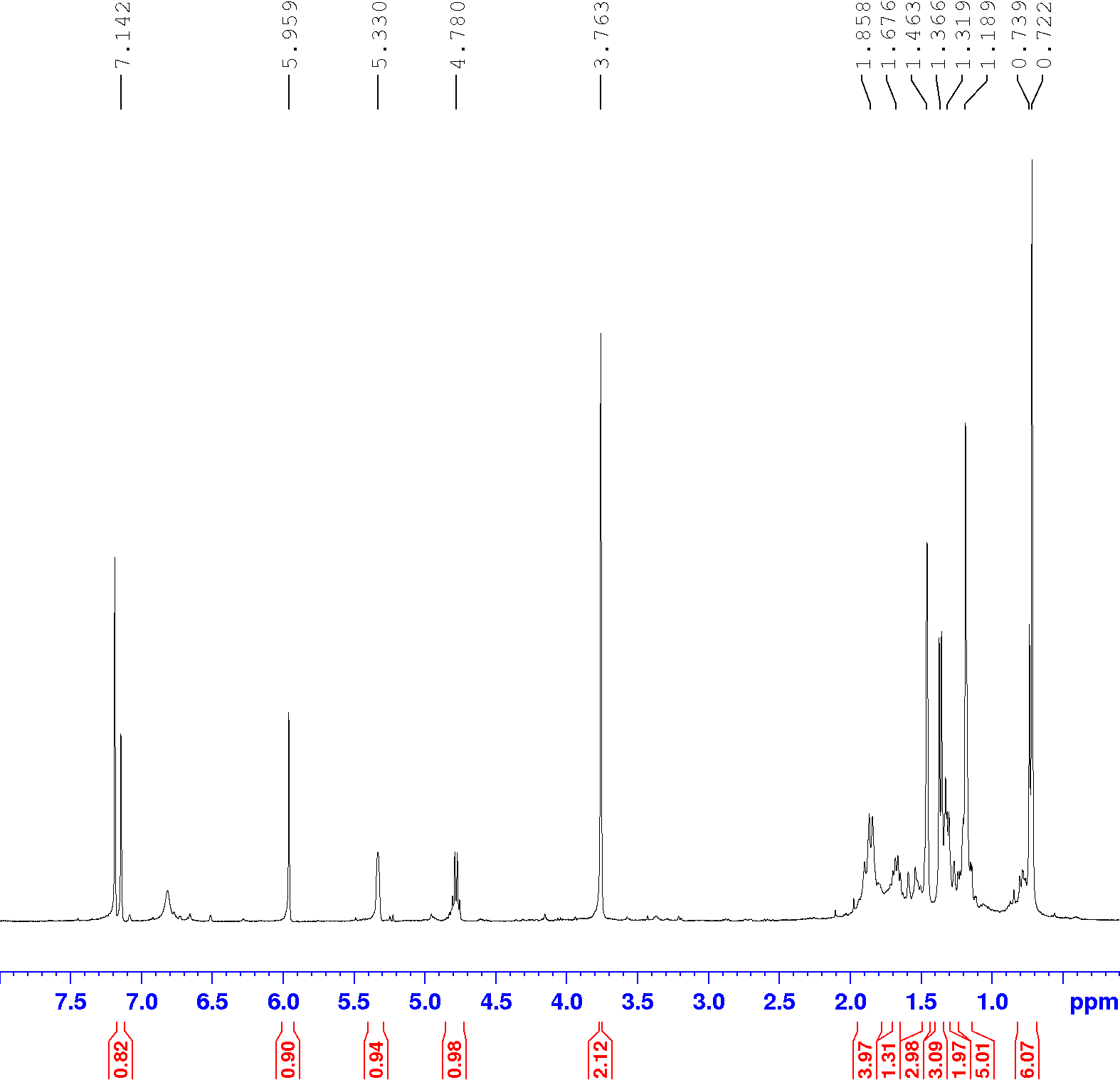


Figure S5. ^1^H-NMR spectrum of the novel compound madeirone (1), recorded at 400 MHz in CDCl_3_ with TMS as internal reference.


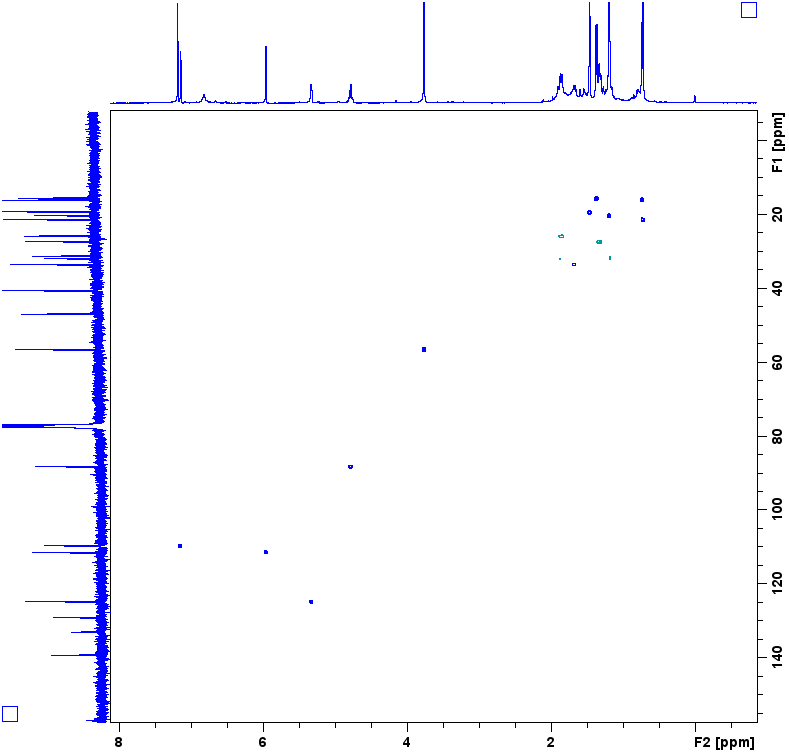


Figure S6. HSQC spectrum of the novel compound madeirone (1), in CDCl_3_ with TMS as internal reference.


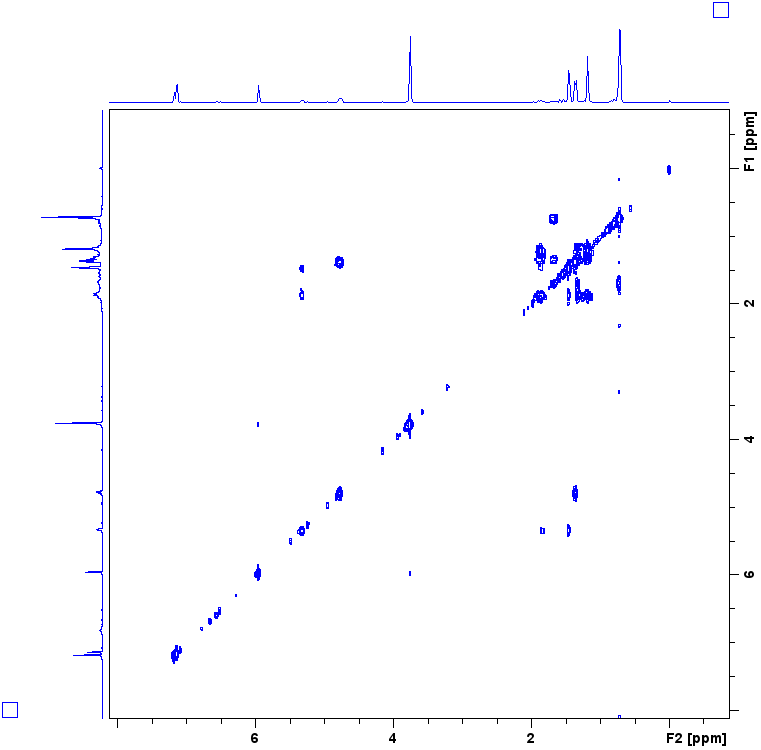


Figure S7. COSY spectrum of the novel compound madeirone (1), recorded at 400 MHz in CDCl_3_ with TMS as internal reference.


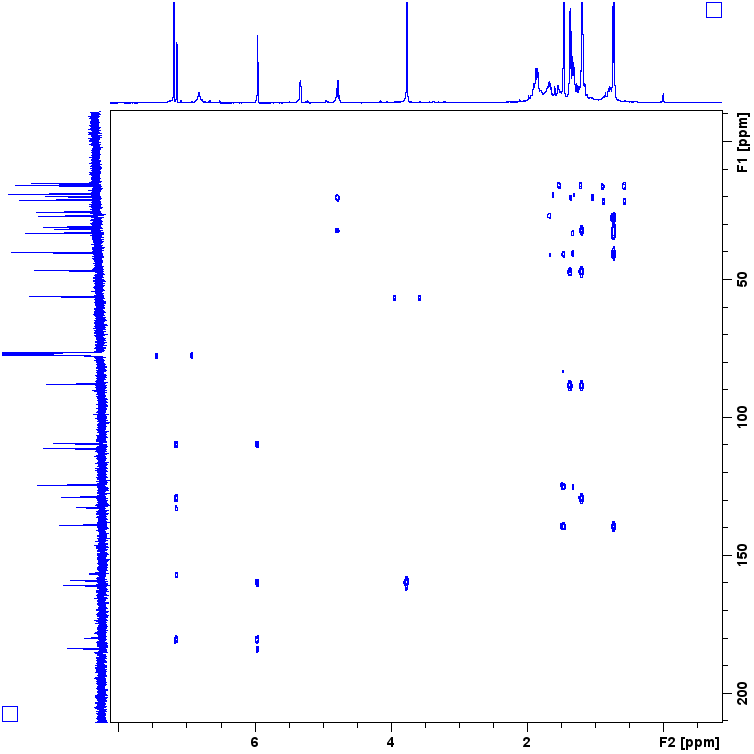


Figure S8. HMBC spectrum of the novel compound madeirone (1) in CDCl_3_ with TMS as internal reference.


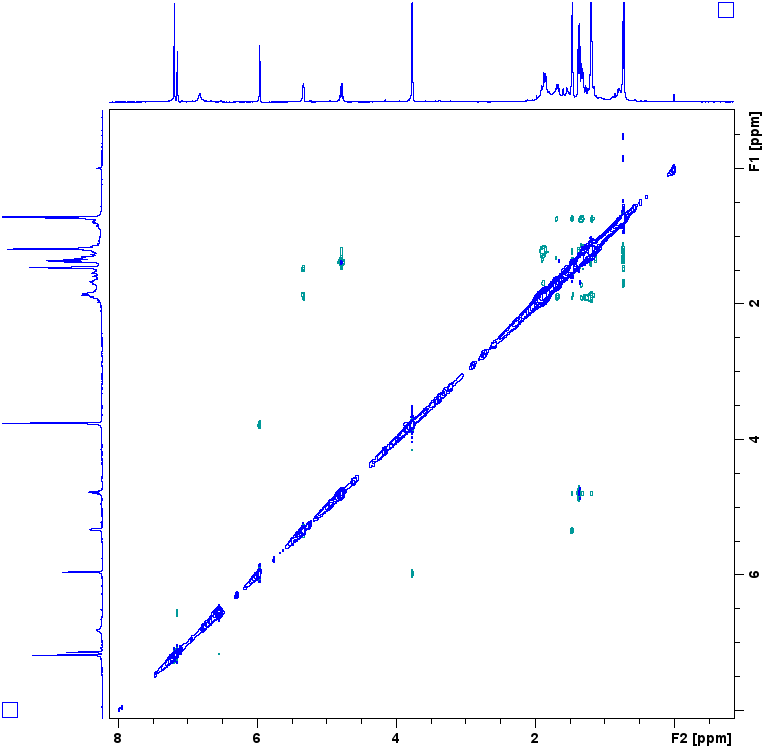


Figure S9. NOESY spectrum of the novel compound madeirone (1) in CDCl_3_ with TMS as internal reference.


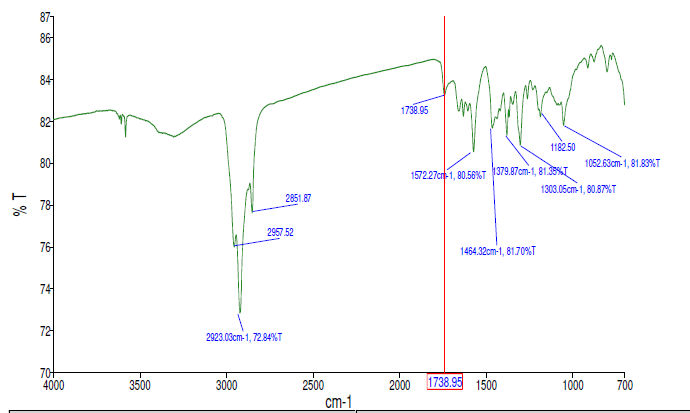


**Figure S10.** IR spectrum of neomarinone (**2**)


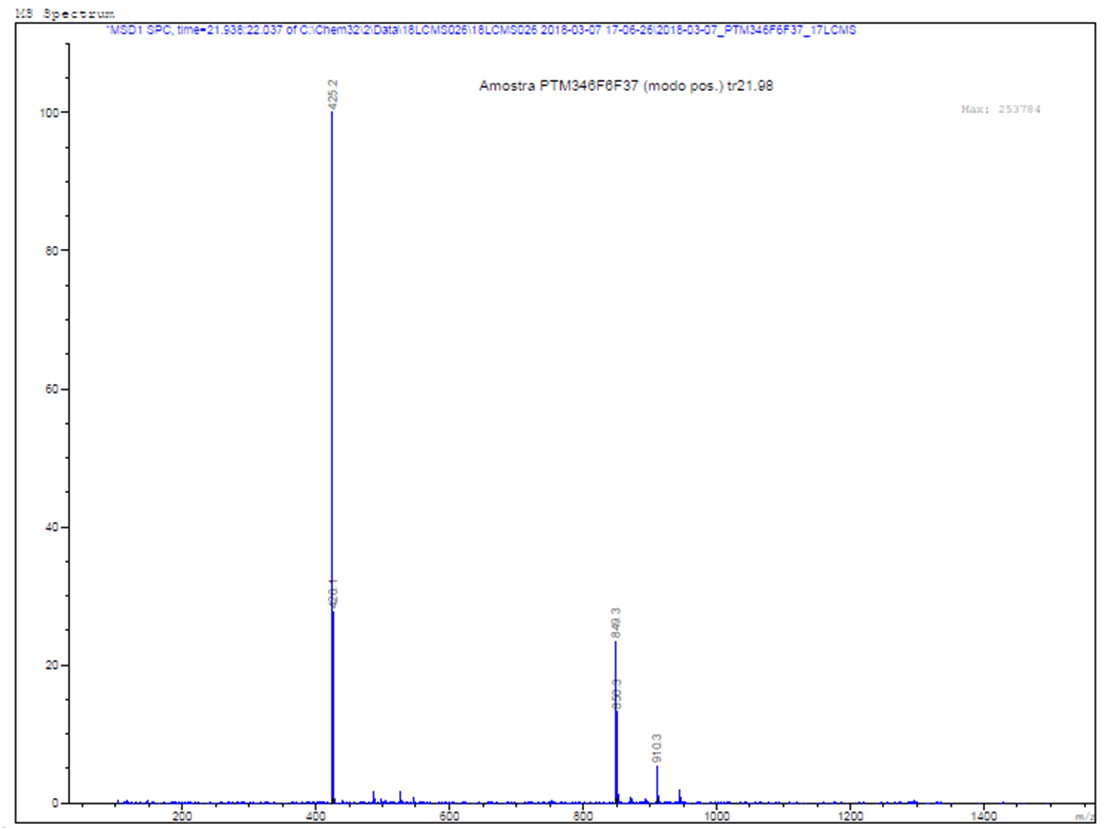


**Figure S11.** Mass spectrum (*m/z*) of neomarinone (**2**) in positive mode. Fragmentation pattern: 425.2 [M+H]^+^, 849.3 [2M+H]^+^.


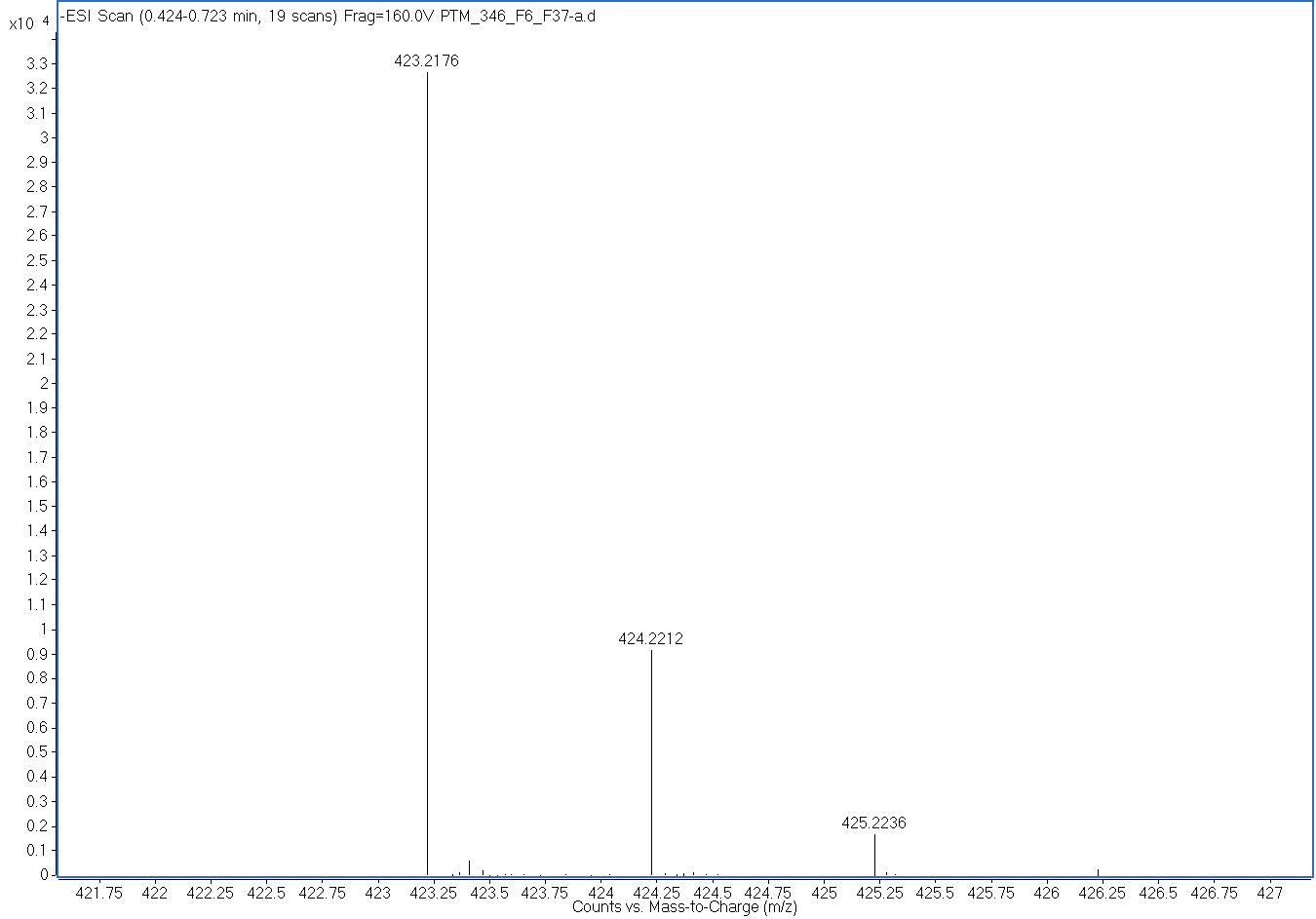


**Figure S12.** High resolution mass spectrum (*m/z*) of neomarinone (**2**), in negative mode. Fragmentation pattern: 423.22 [M-H]^-^.


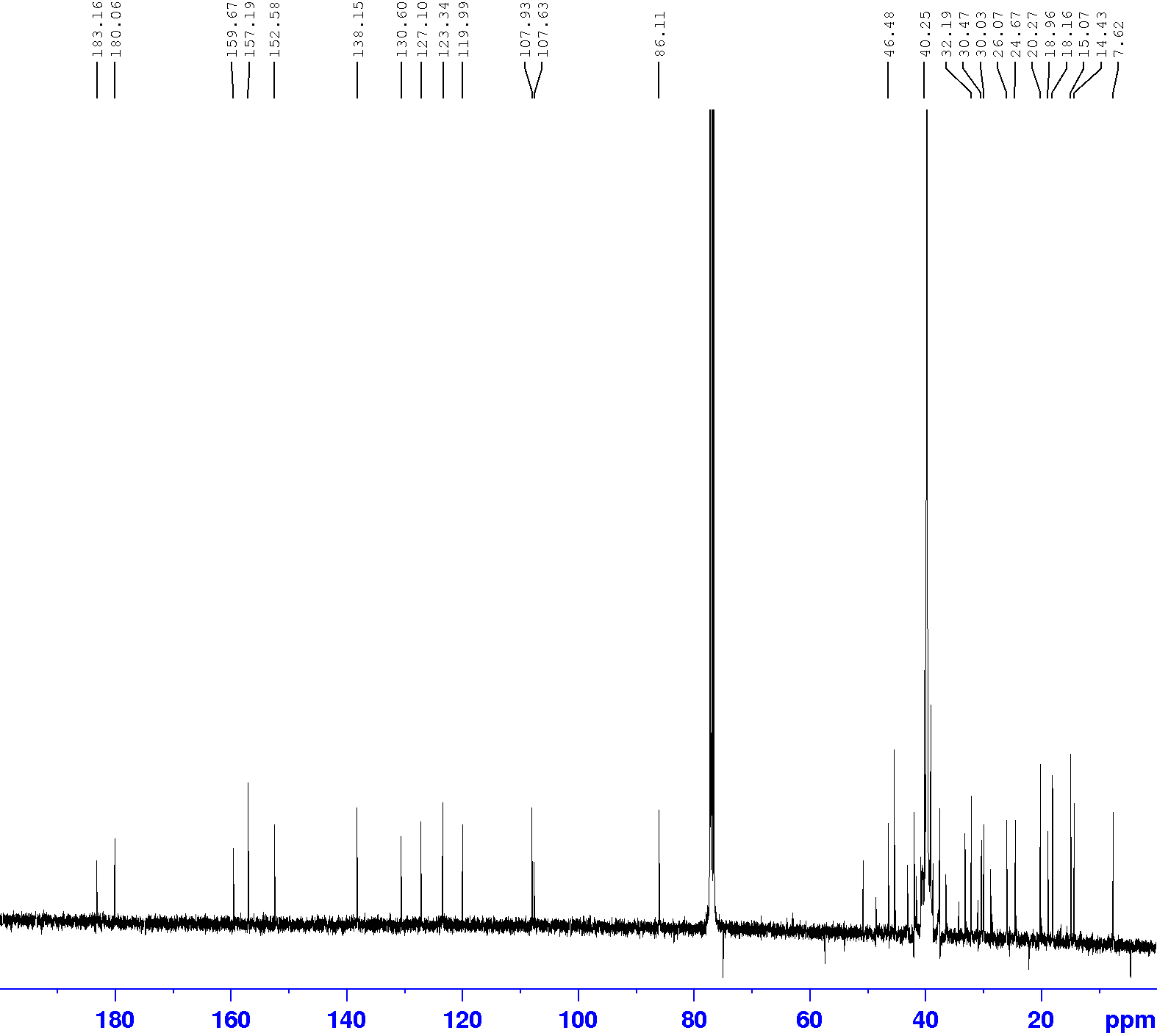


Figure S13. ^13^C-NMR spectrum of neomarinone (2), recorded at 100 MHz in DMSO-d6 with TMS as internal reference.


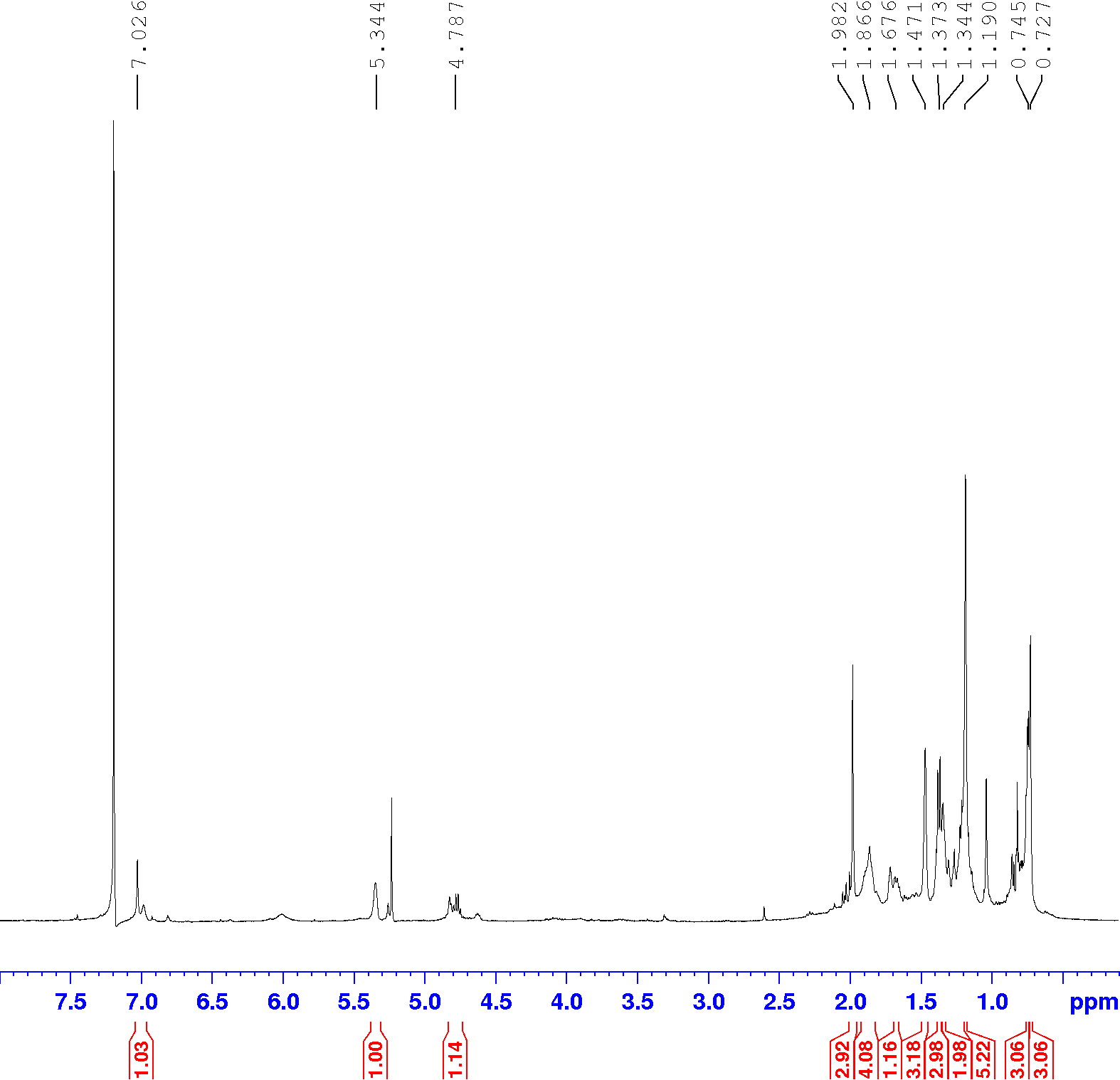


Figure S14. ^1^H-NMR spectrum of neomarinone (2), recorded at 400 MHz in DMSO-d6 with TMS as internal reference. At 7.26 ppm residual CDCl_3_ (solvent) can be seen.


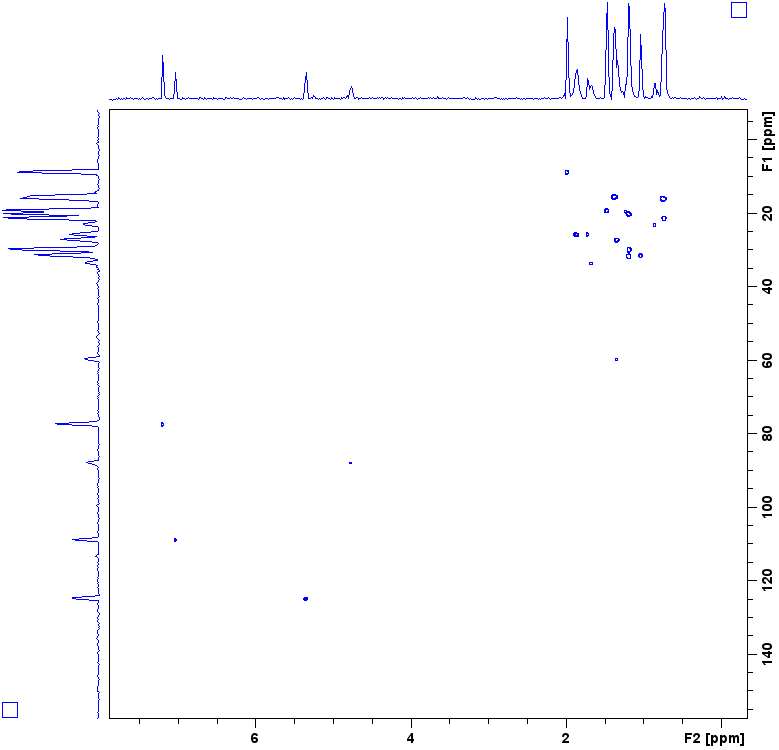


**Figure S15**. HSQC spectrum of neomarinone (**2**) in DMSO-d6 with TMS as internal reference.


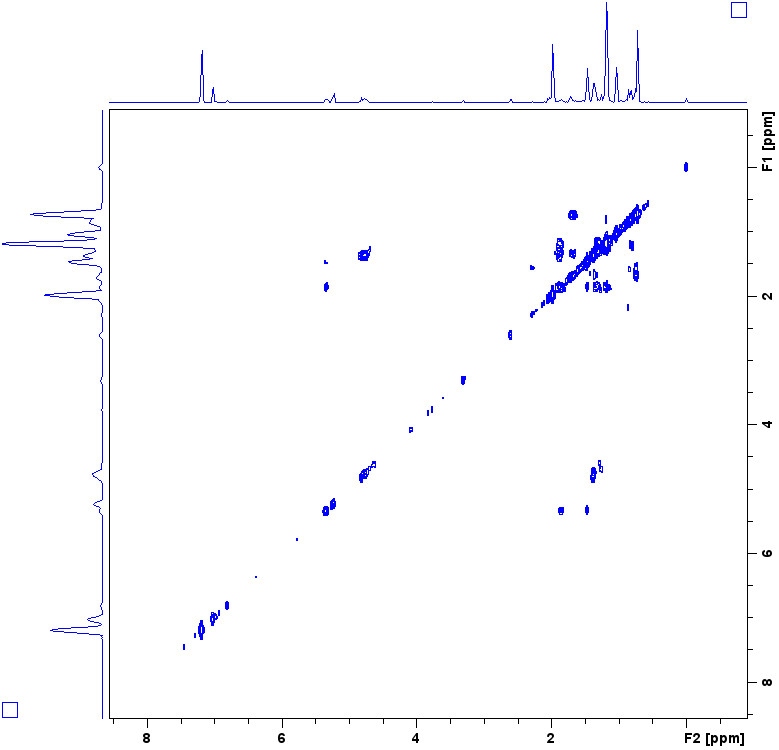


**Figure S16**. COSY spectrum of neomarinone (**2**) recorded at 400 MHz in DMSO-d6 with TMS as internal reference.


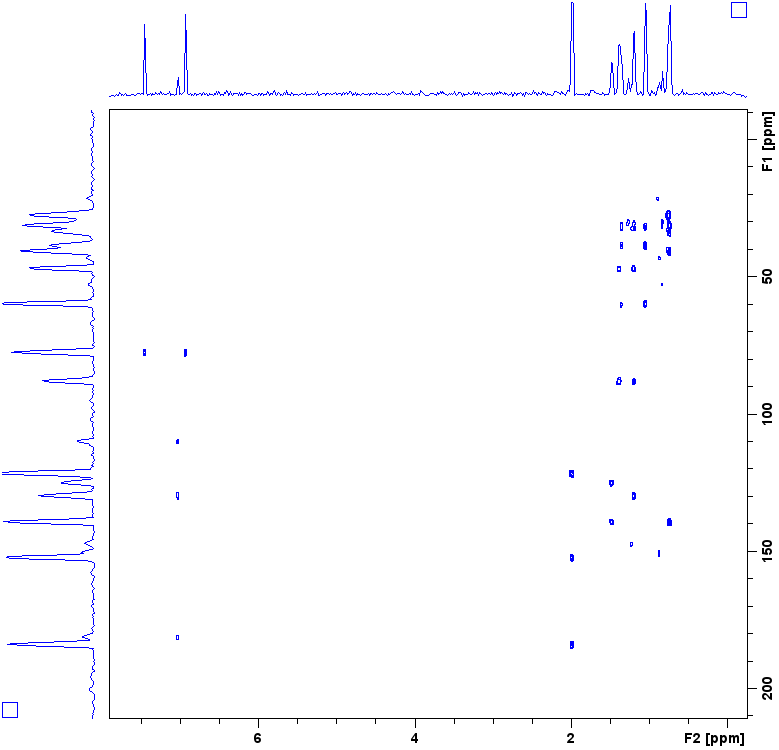


**Figure S17**. HMBC spectrum of neomarinone (**2**) in DMSO-d6 with TMS as internal reference.


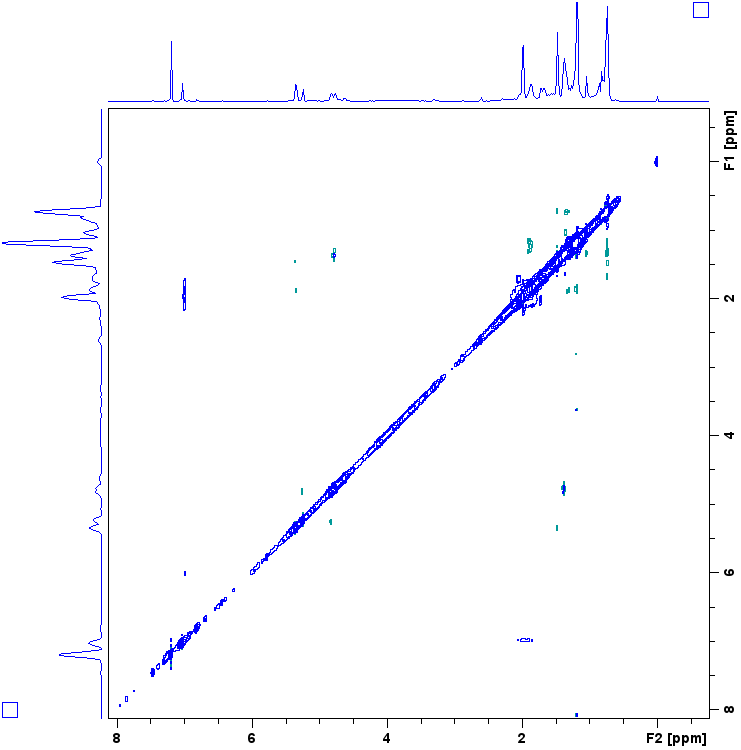


Figure S18. NOESY spectrum of neomarinone (2) in DMSO-d6 with TMS as internal reference.
